# Deficient adaptation to centrosome duplication defects in neural progenitors causes microcephaly and subcortical heterotopias

**DOI:** 10.1172/jci.insight.146364

**Published:** 2021-08-23

**Authors:** José González-Martínez, Andrzej W. Cwetsch, Diego Martínez-Alonso, Luis R. López-Sainz, Jorge Almagro, Anna Melati, Jesús Gómez, Manuel Pérez-Martínez, Diego Megías, Jasminka Boskovic, Javier Gilabert-Juan, Osvaldo Graña-Castro, Alessandra Pierani, Axel Behrens, Sagrario Ortega, Marcos Malumbres

**Affiliations:** 1 Cell Division and Cancer group, Spanish National Cancer Research Centre (CNIO), Madrid, Spain.; 2 Imagine Institute of Genetic Diseases, University of Paris, Paris, France.; 3 Institute of Psychiatry and Neuroscience of Paris, INSERM U-1266, University of Paris, Paris, France.; 4 Adult Stem Cell Laboratory, The Francis Crick Institute, London, United Kingdom.; 5 Confocal Microscopy Unit and; 6 Electron Microscopy Unit, CNIO, Madrid, Spain.; 7 University of Paris, NeuroDiderot, Inserm, Paris, France.; 8 Bioinformatics Unit, CNIO, Madrid, Spain.; 9 Faculty of Life Sciences, King’s College London, Guy’s Campus, London, United Kingdom.; 10 Mouse Gene Editing Unit, CNIO, Madrid, Spain.

**Keywords:** Cell Biology, Development, Cell cycle, Genetic diseases, Neurodevelopment

## Abstract

Congenital microcephaly (MCPH) is a neurodevelopmental disease associated with mutations in genes encoding proteins involved in centrosomal and chromosomal dynamics during mitosis. Detailed MCPH pathogenesis at the cellular level is still elusive, given the diversity of *MCPH* genes and lack of comparative in vivo studies. By generating a series of CRISPR/Cas9-mediated genetic KOs, we report here that — whereas defects in spindle pole proteins (ASPM, *MCPH5*) result in mild MCPH during development — lack of centrosome (CDK5RAP2, *MCPH3*) or centriole (CEP135, *MCPH8*) regulators induces delayed chromosome segregation and chromosomal instability in neural progenitors (NPs). Our mouse model of MCPH8 suggests that loss of CEP135 results in centriole duplication defects, TP53 activation, and cell death of NPs. *Trp53* ablation in a Cep135-deficient background prevents cell death but not MCPH, and it leads to subcortical heterotopias, a malformation seen in MCPH8 patients. These results suggest that MCPH in some MCPH patients can arise from the lack of adaptation to centriole defects in NPs and may lead to architectural defects if chromosomally unstable cells are not eliminated during brain development.

## Introduction

**Figure 1:**
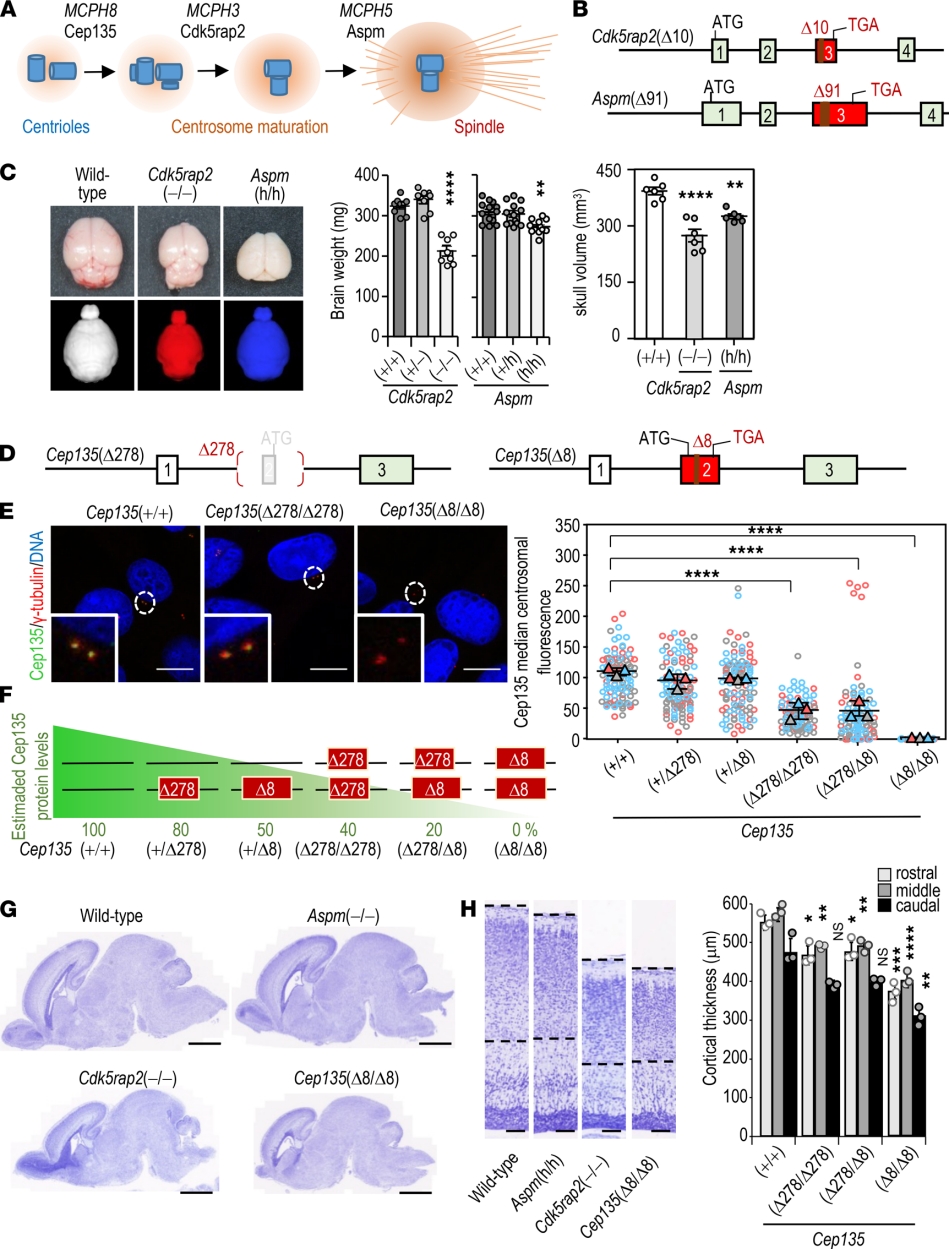
A comparative analysis of MCPH models in the mouse. (**A**) Role of MCPH8 proteins in centriole duplication, centrosome maturation, and spindle pole formation, respectively. (**B**) Generation of the *Cdk5rap2*(–)(Δ10) and *Aspm*(h)(Δ19) alleles by CRISPR/Cas9 editing in mouse embryos. (**C**) Representative images of brains derived from P30 mice (upper) and skull volumes as scored by CT-scan analysis (lower). Histograms to the right depict the quantifications of brain weight of P30 *Cdk5rap2*-mutant (*n* = 8) and *Aspm*-mutant (*n* = 12) mice and the skull volume at P30 (*n* = 6/group). (**D**) Generation of the *Cep135*(Δ278) and *Cep135*(Δ8) alleles. The *Cep135*(Δ278) allele lacks the initial ATG site. The Δ8 allele generates of a premature stop codon in exon 3. (**E**) Confocal imaging of centriolar loading of CEP135 (green) in MEFs with different combinations of *Cep135*(Δ278)-mutant and *Cep135*(Δ8)-mutant alleles. γ-Tubulin, red; CEP135, green; DNA, blue. Stained with DAPI. Scale bars: 10 μm. The superplot to the right shows the quantification of CEP135 centrosomal protein levels in cells with the indicated genotypes. Each color represents data from a different mouse. Horizontal bars depict the mean; ***P < 0.001 (unpaired *t* test with Welsh correction). (**F**) A summary of the effect of the different *Cep135* alleles in the centrosomal levels of CEP135. (**G**) Representative micrographs of sagittal brain sections stained with Nissl at P0 in neonate *Aspm*, *Cdk5rap2*, and *Cep135* mutants. Scale bars: 1 mm. (**H**) Histological Nissl staining of P0 cortices from the indicated mice (left panels). Scale bars: 100 μm. Quantification of cortical thickness in rostral, medial, and caudal aspects of P0 *Cep135*-mutant mice versus *Cep135*(+/+) controls (histogram to the right). Data in **C** and **H** represent mean ± SEM from 3 different mice or embryos; *P < 0.05; **P < 0.01; ***P < 0.001; ****P < 0.0001; 1-way ANOVA with Tukey’s multiple comparisons.****

Autosomal recessive primary microcephaly (MCPH) is a congenital brain disorder characterized by a reduction in head circumference linked to a striking decrease in brain volume (from –3 to –13 SDs). These changes are typically not linked with gross anomalies of brain architecture and associate with a primary and selective defect in the production of neurons during development. Several genes mutated in MCPH patients have been identified so far (*MCPH1*-*25*), including genes encoding proteins associated with centriole biology or the mitotic spindle such as *ASPM* (MCPH5) and *WDR62* (MCPH2), the 2 most commonly mutated MCPH genes (1–4) (Figure 1A). Centrioles are barrel-shaped, membrane-less organelles that behave as the major microtubule organizing centers (MTOCs) generating key cellular structures such as centrosomes, cilia, and flagella. Structurally, centrioles consist of 9 microtubule triplets organized around a cartwheel from which 9 protein spokes emanate radially, conferring a 9-fold symmetric conformation that stabilizes the structure (5). These structures recruit several proteins to polymerize a robust pericentriolar matrix (PCM), thereby allowing the generation of a bipolar spindle that ensures the correct segregation of chromosomes to both daughter cells, thus avoiding chromosomal instability. Deregulation of centrosome dynamics typically results in a variety of cell division defects, ultimately affecting neural development (4).

Centriole duplication relies on the formation of a newborn procentriole in an orthogonal angle using the preexisting centriole as a biogenesis platform (5). The core centriole duplication toolbox required for this process includes enzymatic components such as Polo-like kinase 4 (PLK4), which acts as a key regulator of centriole biogenesis (6, 7), as well as structural components such as SAS4 (MCPH6, also known as CPAP or CENPJ) (8), SAS6 (MCPH14) (9), or CEP135 (MCPH8) (10). CEP135 was originally identified as a component of the cartwheel, with critical scaffold function in centriole structure and biogenesis (10). Pioneer studies in *Drosophila* suggested minor defects in centriole duplication after *Cep135* knockdown (11–14), as well as mild perturbations of centriole structure in *Cep135*-mutant spermatocytes (14). *Cep135* knockdown in chicken cell lines does not significantly alter centriole structure and organization, nor centriole biogenesis or cell proliferation, and only mild centrosome duplication defects arise (15). In contrast, genetic knockdown by RNA interference of *CEP135* in human cell lines induced significant centriole defects, altered spindle assembly and aberrant centriole structure (16). Interestingly, genetic analysis of a cohort of MCPH patients identified a homozygous single bp deletion in exon 8 of human *CEP135*, which resulted in a frameshift that produced a premature stop codon (17). Patients bearing this mutation present severe MCPH accompanied by subcortical heterotopia (cell clumps between the lateral ventricles and the cortex; ref. 18) — a finding not common in other MCPH patients and whose pathogenic origin is unclear.

In this work, we have generated a series of MCPH models with specific alterations in centriole (CEP135), centrosome (CDK5RAP2), and spindle pole (ASPM) proteins by using CRISPR/Cas9-mediated direct editing of mouse embryos. Whereas *Aspm* ablation results in minor defects in cortical development, lack of *Cdk5rap2* or *Cep135* results in significant primary MCPH accompanied by centrosome duplication, chromosomal instability, and a TP53-associated antiproliferative response in the developmental cortex. Genetic ablation of TP53 prevents apoptosis during midgestation but does not prevent MCPH and leads to heterotopia in late development, a relatively rare finding in MCPH (including MCPH8), as well as in patients with seizures and intellectual disability.

## Results

### A comparative analysis of MCPH models in the mouse.

We first generated mouse models of MCPH3 and MCPH5 (Figure 1A) by introducing loss-of-function mutations in the murine *Cdk5rap2* and *Aspm* genes (Figure 1B). In agreement with previous mouse models for these pathologies (19–21), lack of *Cdk5rap2* (*Cdk5rap2*[–] allele) and partial lack of *Aspm* (*Aspm*[h] allele) (Supplemental Figure 1A; supplemental material available online with this article; https://doi.org/10.1172/jci.insight.146364DS1) resulted in significant defects in brain weight and volume at P30 (Figure 1C and Supplemental Figure 1B). Both *Cdk5rap2*(–/–) and *Aspm*(h/h) mice displayed reduced maturation of germ cells in the testis (Supplemental Figure 1, C and D) as reported previously (19, 20). We also observed a previously undefined defect in retinal development causing microphtalmia in *Cdk5rap2*-deficient embryos (Supplemental Figure 1E), in agreement with cataracts and ocular abnormalities observed in MCPH3 patients (22, 23). Cortical thickness was reduced in both models at E14.5 and adult (P30) neocortices, with a stronger phenotype in the absence of *Cdk5rap2* (Supplemental Figure 1, F and G).

Since a previous model of MCPH8 did not exist, we next decided to generate mice with loss-of-function mutations in *Cep135*. We targeted the second exon of murine *Cep135* using 2 single-guide RNAs (sgRNAs) generating a mutant sequence with a 278 bp deletion (*Cep135*[Δ278] allele lacking the initial ATG site) and an 8 bp deletion (*Cep135*[Δ8] allele) (Figure 1D and Supplemental Figure 2A). *Cep135*(Δ278/Δ278) mouse embryonic fibroblasts (MEFs) displayed reduced protein levels at the centrosome, whereas no detectable CEP135 was present in *Cep135*(Δ8/Δ8) cells (Figure 1E). Combination of these alleles in either homozygosity or heterozygosity allowed the generation of 6 cohorts of *Cep135*-mutant mice with different amounts of CEP135 protein, ranging from WT levels to intermediate levels in *Cep135*(Δ278/Δ278) homozygous mutants and *Cep135*(Δ278/Δ8) heterozygous mice, and no detectable protein in the *Cep135*(Δ8/Δ8) model (Figure 1F).

These alleles allowed a comparison between *Cep135* gene dosage and the phenotype arising from different CEP135 protein levels in vivo. Both *Cep135*(Δ278/Δ278) and *Cep135*(Δ278/Δ8) newborns (P0) displayed mild MCPH (~24.5% and 24.7% reduction in cortical area, respectively), whereas homozygous *Cep135*(Δ8/Δ8) P0 mice showed severe MCPH, as showed by a reduced neocortical area (48.5% cortical area reduction; Supplemental Figure 2, B–E, and Figure 1, G and H) and reduced neocortical thickness in the rostral, medial, and caudal aspects of the neonatal neocortices (Figure 1G). The reduction in head and brain weight was more pronounced than the reduction in body weight (Supplemental Figure 2E), suggesting that *Cep135* depletion may promote MCPH in a gene dose-dependent manner in mice. Full depletion of CEP135 in the *Cep135*(Δ8/Δ8) model (Figure 1E) resulted in perinatal lethality, with all mutant mice dying within a few hours after birth. Histological examination of *Cep135*-null i.p. organs revealed simplified and underdeveloped intestinal and stomach mucosae, with no evidence of recent lactation, immature and underdeveloped lungs, and retinal abnormalities (Supplemental Figure 2, F and G). Detailed analysis of the lungs revealed small, immature alveolae with lower levels of Surfactant Protein-C (Supplemental Figure 2H), suggestive of respiratory distress. *Cep135*(+/Δ278) and *Cep135*(+/Δ8) heterozygous mice exhibited no significant differences in the centrosomal levels of CEP135 (Figure 1E) and, accordingly, no MCPH, body size reduction, or other phenotypic defects were observed (data not shown).

### Lack of Cep135 induces multiple defects in centriole dynamics and chromosomal instability.

**Figure 2:**
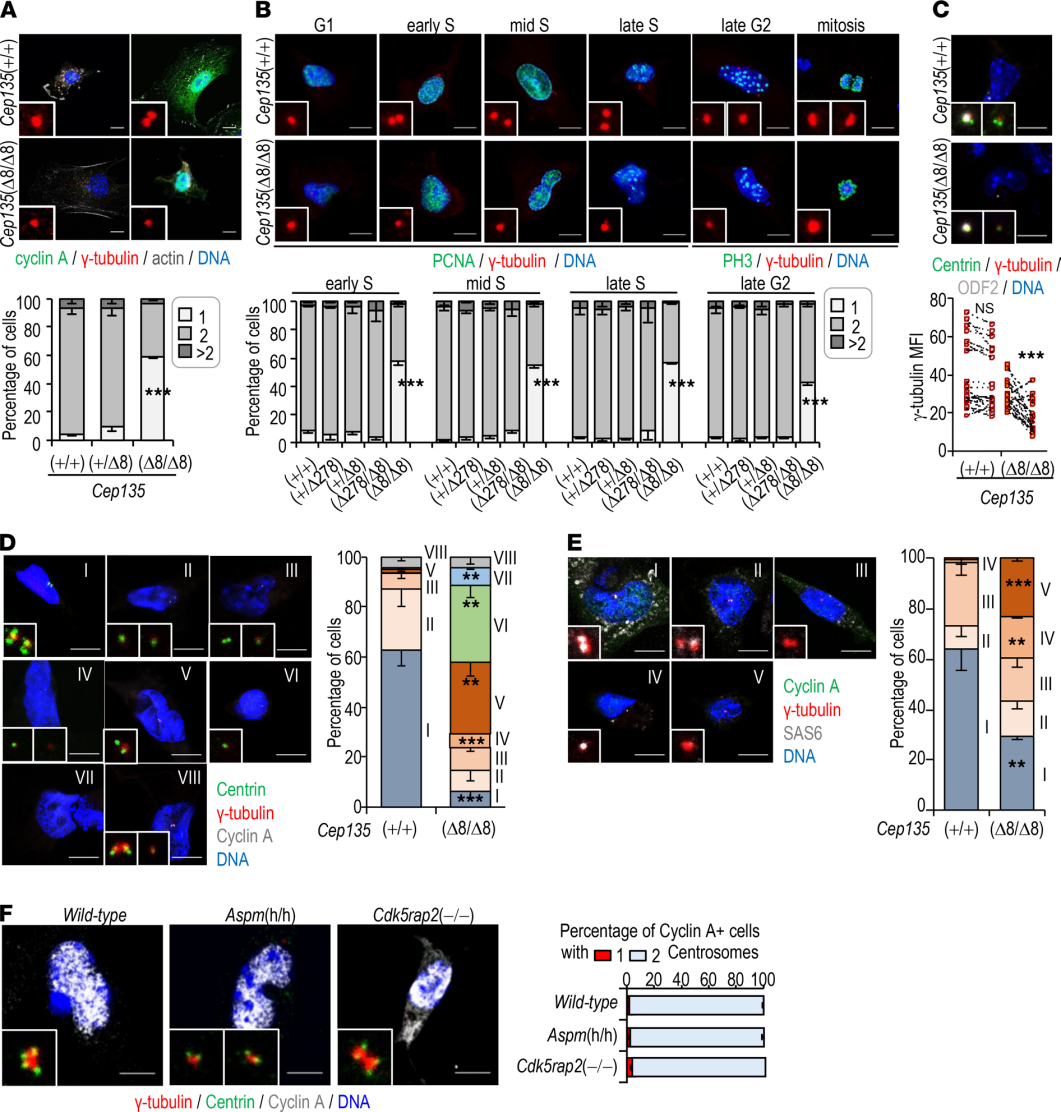
Centrosome dynamics defects in Cep135-deficient fibroblasts. (**A**) Confocal imaging of E14.5 *Cep135*-mutant and control MEFs stained with the indicated markers. The histogram shows the percentage of cells with 1, 2, or more than 2 γ-tubulin spots. (**B**) Immunostaining of MEFs with the indicated markers. The bottom histograms show the percentage of cyclin A+ cells with 1, 2, or more than 2 γ-tubulin spots in the different phases of the cell cycle, as determined by PCNA and PH3 staining. (**C**) Immunostaining with antibodies against centrin (green), γ-tubulin (red), and ODF2 (cenexin, a marker of the mother centrosome; gray), and quantification of γ-tubulin mean fluorescence intensity (MFI). Slashed lines link 2 centrosomes from the same cell; higher slope of the line indicates higher centrosomal asymmetry. (**D**) Confocal imaging of E14.5 *Cep135*(Δ8/Δ8) MEFs. All cells depicted are cyclin A+. Group I: 2 γ-tubulin spots; 2 centrin doublets. Group II: 2 γ-tubulin spots; 2 centrin singlets. Group III: 2 γ-tubulin spots; 1 centrin doublet + 1 centrin singlet. Group IV: 2 γ-tubulin spots; 1 centrin doublet + 1 centrin singlet. Group V: 1 γ-tubulin spot; 1 centrin doublet. Group VI: 1 γ-tubulin spot; 1 centrin singlet. Group VII: acentrosomal. Group VIII: >2 γ-tubulin spots. (**E**) As in **D**. Group I: 2 γ-tubulin spots; 2 Sas6 singlets. Group II: 2 γ-tubulin spots; 1 Sas6 singlet + no Sas6. Group III: 2 γ-tubulin spots; no Sas6 – no Sas6. Group IV: 1 γ-tubulin spot; 1 Sas6 singlet. Group V: 1 γ-tubulin spot; no Sas6. (**F**) Confocal imaging of E14.5 MEFs and percentage of cyclin A+ cells with 1 or 2 γ-tubulin spots. Scale bars: 10 μm (**A**–**F**). Data are mean ± SEM from 3 cultures from 3 E14.5 embryos; *n* > 150 cells/condition; **P < 0.01; ***P < 0.001; 1-way ANOVA with Tukey’s multiple comparisons (**A**,** B**, and** F**) and Student’s *t* test (**C**–**E**).

To gain a deeper insight about the cellular alterations generated by CEP135 loss, we initially screened for centrosome or cytoskeleton defects in CEP135-deficient MEFs. Whereas 2 foci of γ-tubulin (a major component of the pericentriolar material [PCM]) were detected in most control cells positive for cyclin A (a marker of late S-G2 phases of the cell cycle), only 1 γ-tubulin structure was found in the majority of cyclin A^+^ cells derived from *Cep135*-KO embryos (Figure 2A). For a more detailed analysis of cell cycle stages, we made use of proliferating cell nuclear antigen (PCNA), a DNA replication processivity factor whose nuclear pattern changes depending of the period of the S-phase, and phospho-histone H3 (PH3), a modification that correlates with chromosome condensation during late G2 and mitosis. Whereas both control and *Cep135*-mutant cells displayed a single centrosome in G1, coimmunostaining of γ-tubulin with PCNA revealed efficient centrosome duplication in control *Cep135*(+/+) but not in mutant *Cep135*(Δ8/Δ8) cells (Figure 2B). In about 50% of *Cep135-*null cells, a single γ-tubulin spot was present from G1 to late G2, inducing monopolar spindles during mitosis (Figure 2B). Interestingly, the remaining fraction of *Cep135*-KO cells that contained 2 centrosomes presented additional defects, such as centrosomal asymmetry in the maturation of centrosomes when stained for Centrin (a centriolar protein) and the PCM component γ-tubulin. This defect typically affected the daughter, newly generated centrosome, and not the mother centrosome, which is characterized by the presence of Outer Dense Fiber Protein 2 (ODF2; Figure 2C).

Combined immunofluorescence for centrin and γ-tubulin in cyclin A–positive cells indicated that centrosome duplication was compromised in *Cep135*(Δ8/Δ8) cells (Figure 2D, group V). In addition, these mutant cultures were enriched in cells with only 1 centrosome and 1 centriole, suggestive of both centrosome duplication and centriole assembly defects (Figure 2D, group VI), as well as in cells lacking centrosomes (acentrosomal, group VII). Similar results were obtained in late G2 PH3^+^ cells, confirming that centriole defects caused by CEP135 depletion were also sustained during the rest of the cell cycle (Supplemental Figure 3A). Immunostaining of S/G2 cells (Cyclin A^+^) for PCM (γ-tubulin) and cartwheel proteins (Sas6) revealed a significant increase in cells with only 1 centrosome devoid of cartwheel (Figure 2E; group V), suggesting additional defects in cartwheel assembly. Interestingly, MEFs derived from *Aspm-* and *Cdk5rap2*-mutant embryos did not present significant centrosome duplication defects (Figure 2F), suggesting that centrosome duplication defects are a specific feature generated by the absence of CEP135.

The presence of 2 properly matured and separated centrosomes is critical for the establishment of a bipolar spindle and correct chromosome segregation. Time-lapse microscopy analysis of *Cep135*(Δ278/Δ278) or *Cep135*(Δ278/Δ8) cultures showed no alteration in the duration of mitosis. However, homozygous cells for the *Cep135*(Δ8) mutation displayed significantly increased duration of mitosis, in agreement with abundant monopolar spindles (Supplemental Figure 3, B and C). These defects were accompanied by increased nuclear volume, DNA content (4n and more than 4n) and aneuploidy in a *Cep135* gene dose-dependent manner (Supplemental Figure 3, D–F). These results suggest that loss of CEP135 in the *Cep135*(Δ8/Δ8) model leads to defective centriole dynamics, ultimately inducing chromosomal instability, at least in fibroblasts.

### Perturbed centrosomal and mitotic dynamics in Cep135-deficient neural progenitors.

**Figure 3:**
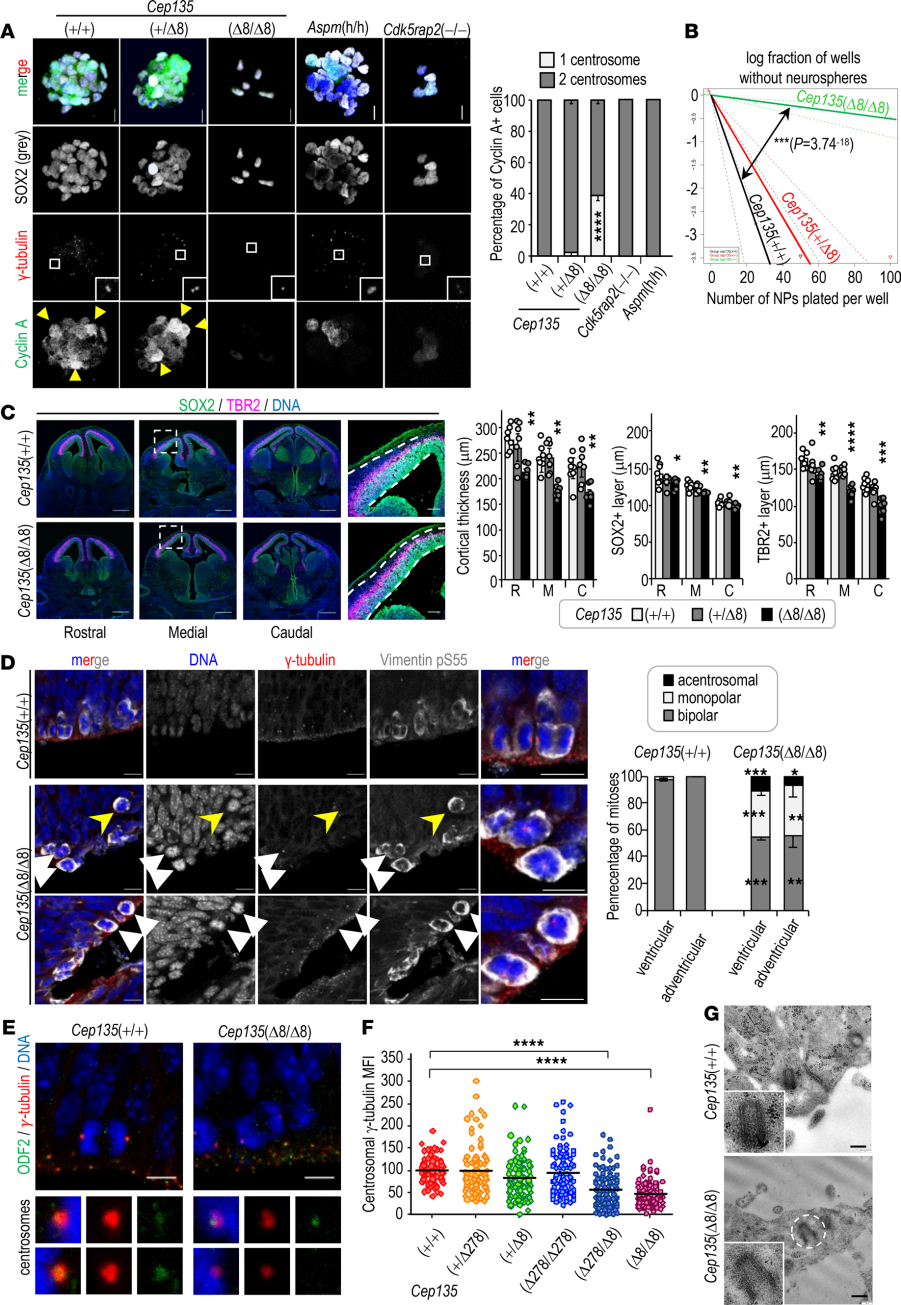
Self-renewal ability and centrosome dynamics in Cep135-mutant neural progenitors. (**A**) Confocal imaging of neurospheres derived from E14.5 embryonic cortices stained with the indicated antibodies, and percentage of cyclin A+ cells with 1 or 2 centrosomes. (**B**) Self-renewal ability of neural progenitors as determined by limiting dilution assays. (**C**) Confocal imaging of cryosections of the rostral, medial, and caudal aspects of E14.5 embryonic brains with the indicated markers. Scale bar: 1 mm (left) and 100 μm (insets). Quantification of the thickness (left), SOX2^+^ cells (middle), and TBR2^+^ cells (right) in rostral (R), medial (M), and caudal (C) regions. (**D**) Immunofluorescence with the indicated antibodies in developing E14.5 neocortices. Control samples show typical bipolar spindles, whereas monopolar spindles (white arrowheads in middle panels), acentrosomal spindles (yellow arrowheads), and asymmetric centrosomes (arrowheads in bottom panels) are observed in *Cep135*-mutant samples. Scale bars: 25 μm. The histogram shows the quantification of polarity in mitotic spindles (monopolar, bipolar, or acentrosomal) in ventricular or abventricular mitoses of E14.5 neocortices. (**E**) Immunostaining with the indicated antibodies of anaphases in the ventricular surface of E14.5 *Cep135*-mutant and control embryos. Higher-magnification images of mitotic centrosomes are displayed in the bottom panels. Scale bar: 10 μm. (**F**) Mean fluorescence intensity (MFI; arbitrary units) of γ-tubulin at the centrosome from NPs of the indicated genotypes. ****P < 0.0001 (unpaired *t* test with Welsh correction). (**G**) Transmission electron micrographs showing representative pictures of centrioles contained in the first layer of APs of the ventricular surface. Scale bars: 200 nm. Scale bars: 10 μm (**A**,** D**, and **E**). Arrowheads indicate Cyclin A+ cells (**A**), TP53^+^ cells (**B**), or apoptotic cells (**C**). In **A**–**D**, data are mean ± SEM. **P < 0.01; ***P < 0.001; ****P<0.0001; 1-way ANOVA with Tukey’s multiple-comparison test (**A**,** C**, and **F**), χ^2^ (**B**), Student’s *t* test (**D**).

We next examined the effect of lack of CEP135 in cortical neural progenitors (NPs) isolated from E14.5 neocortices and cultured to generate incipient neurospheres. In agreement with the observations in fibroblasts, *Cep135*-deficient neurospheres displayed centrosome duplication defects, whereas this effect was not evident in *Cdk5rap2*- or *Aspm*-deficient progenitors (Figure 3A). Lack of *Cep135* also correlated with a significant defect in their self-renewal capacity, as scored by the limiting dilution assays (Figure 3B). Unfortunately, these defects and the reduced viability of these mutant cells (see below) did not allow to establish cultures of *Cep135*(Δ8/Δ8) NPs for further assays of centrosome dynamics in vitro.

We therefore looked for centrosome alterations in vivo. The reduced cortical thickness observed at birth (Figure 1) was also present in these mutant mice at E14.5 and E17.5, and it specifically affected the SOX2^+^ apical progenitor (AP) and the TBR2^+^ intermediate progenitor (IP) cell populations within the developing neocortex (Figure 3C and Supplemental Figure 4, A–D). No significant differences were observed in the orientation of the mitotic spindle in dividing cells of the AP layer in *Cep135*-deficient embryos (Supplemental Figure 4E). However, while most mitoses were bipolar in *Cep135*(+/+) embryos, *Cep135*(Δ8/Δ8) embryonic sections revealed frequent mitotic defects, including monopolar or acentrosomal mitotic spindles, in addition to apoptotic figures (Figure 3D and Supplemental Figure 4F). Maturation of centrosomes was impaired as detected by overall reduced γ-tubulin staining in APs of *Cep135*-deficient embryos (Figure 3, E and F). *Cep135*(Δ8/Δ8) APs also presented asymmetric centrosomes with 1 of the 2 centrosomes in the poles with dim or almost absent γ-tubulin signal and asymmetric immunostaining of the distal appendage mother centriole protein ODF2 (Figure 3E). Transmission electron microscopy (TEM) analysis of centrioles in APs showed aberrant axial structure in *Cep135*(Δ8/Δ8) embryos, with lack or abnormal number of microtubule triplets, reduced centriolar diameter in some centriolar structures, and lack of a clear 9-fold symmetric conformation (Figure 3G and Supplemental Figure 4G). These results suggest that alteration of CEP135 protein levels lead to defective centriolar duplication and dynamics in NPs, accompanied by MCPH.

### A TP53 response in Cep135-deficient NPs.

**Figure 4:**
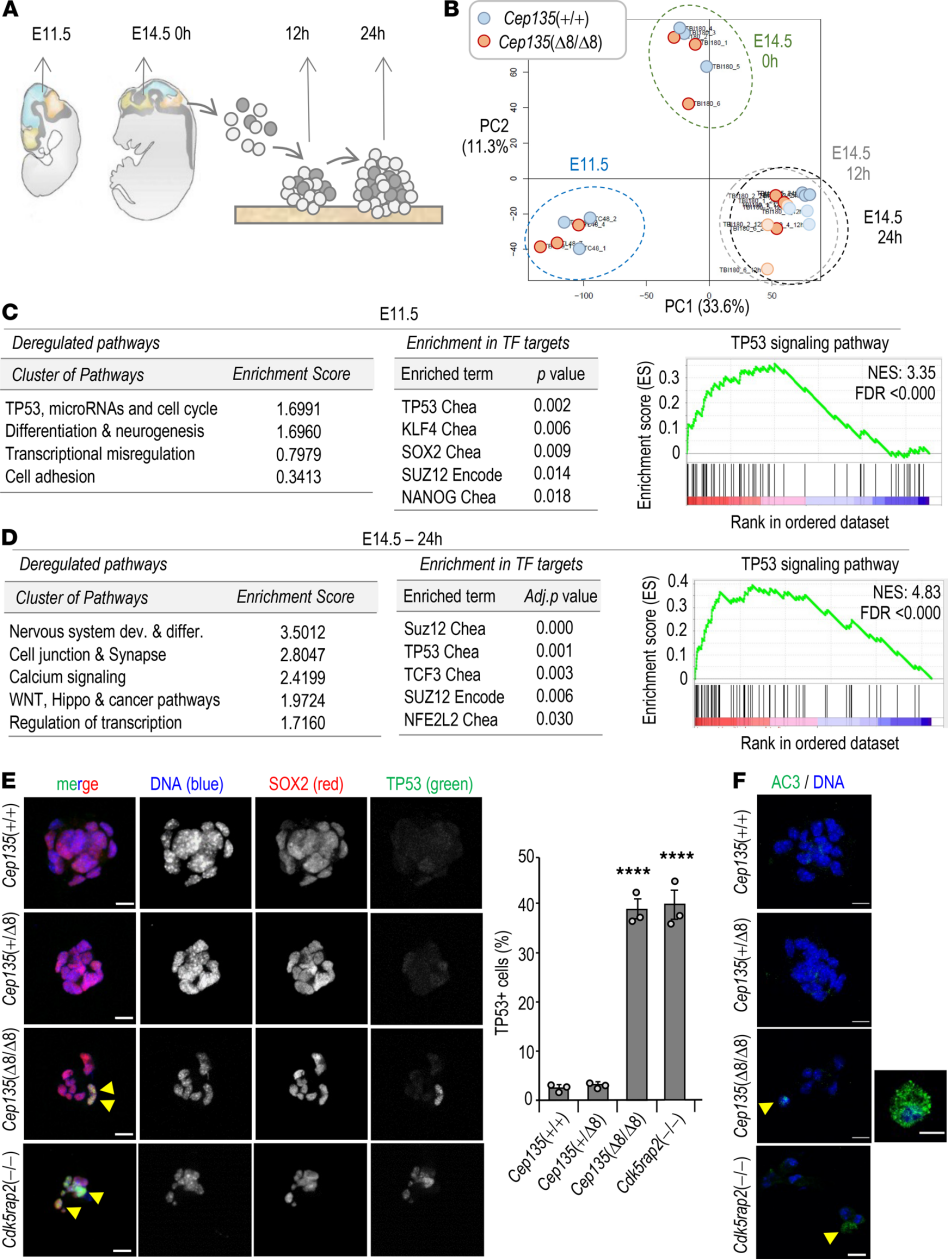
TP53-mediated response to lack of Cep135 in developing brains and cultured neurospheres. (**A**) Schematic representation of the *Cep135*(+/+) and *Cep135*(Δ8/Δ8) samples selected for RNA sequencing analysis, including E11.5 and E14.5 brains, as well as neurospheres from E14.5 brains cultured for 12 and 24 hours. (**B**) Principal component analysis of the transcriptomic profiles from the indicated samples. Values in PC labels show the percentage of explained variance for principal component 1 (PC1) and PC2. (**C**) Major pathways deregulated and enrichment in transcription factor (TF) targets in E11.5 samples. The enrichment in transcripts involved in the TP53 pathway is shown to the right. (**D**) Major pathways deregulated and enrichment in TF targets in E14.5 samples cultured during 24 hours to form neurospheres. The enrichment in transcripts involved in the TP53 pathway is shown to the right. See Supplemental Tables 1–9 for additional details. (**E**) Immunostaining of primary neurospheres from E14.5 embryos with SOX2 and TP53 antibodies. The percentage of TP53^+^ cells is shown to the right. (**F**) Confocal imaging of primary neurospheres stained with antibodies against active caspase 3 (AC3, green). DAPI (DNA) is shown in blue. Scale bars: 10 μm (**E** and **F**). Yellow arrowheads indicate TP53^+^ (**E**) and CC3^+^ cells (**F**). In **E**, data are mean ± SEM. ****P < 0.0001 (1-way ANOVA with Tukey’s multiple-comparison test).

To further gain insight into the molecular pathways deregulated during cortical development in *Cep135*-mutant embryos, we subjected developing cortices from E11.5 and E14.5 *Cep135*(Δ8/Δ8) and control *Cep135*(+/+) embryos to RNA sequencing (RNAseq) analysis. In addition, E14.5 cortices were disaggregated and cultured to form incipient neurospheres, and RNA from these in vitro cultures was analyzed after 12 hours and 24 hours (Figure 4A). Combined analysis of transcriptomic profiles from these samples segregated 3 main groups corresponding to E11.5 cortices, E14.5 cortices, and cultured cells (12- and 24-hour time points; Figure 4B). Differential expression between *Cep135*-null and control samples in each group (Supplemental Figure 5A) suggested a defect in neural differentiation and function in *Cep135*-deficient samples both in vivo at E11.5 (Figure 4C, Supplemental Figure 5B, and Supplemental Table 1) and E14.5 (Supplemental Figure 5C and Supplemental Table 2), as well as in the neurosphere formation assay (Figure 4D, Supplemental Figure 5C, and Supplemental Tables 3 and 4). Interestingly, the TP53 pathway was deregulated in these samples, with significant upregulation of multiple TP53 target genes and a significant enrichment of deregulated transcripts with TP53 binding sites in their promoters (Figure 4, C and D; Supplemental Figure 5C; and Supplemental Tables 5–8). The TP53 target and cell cycle inhibitor *Cdkn1a* (p21^Cip1^) was among the most upregulated genes in these samples (Supplemental Table 9). Immunofluorescence studies in neurosphere cultures detected a significant presence of TP53^+^ cells in CEP135-deficient progenitors, as well as neurospheres cultured from CDK5RAP2-null progenitors (Figure 4E). This signal was accompanied by apoptotic cell death in neurosphere cells, as detected by active caspase 3 (Figure 4F).

**Figure 5:**
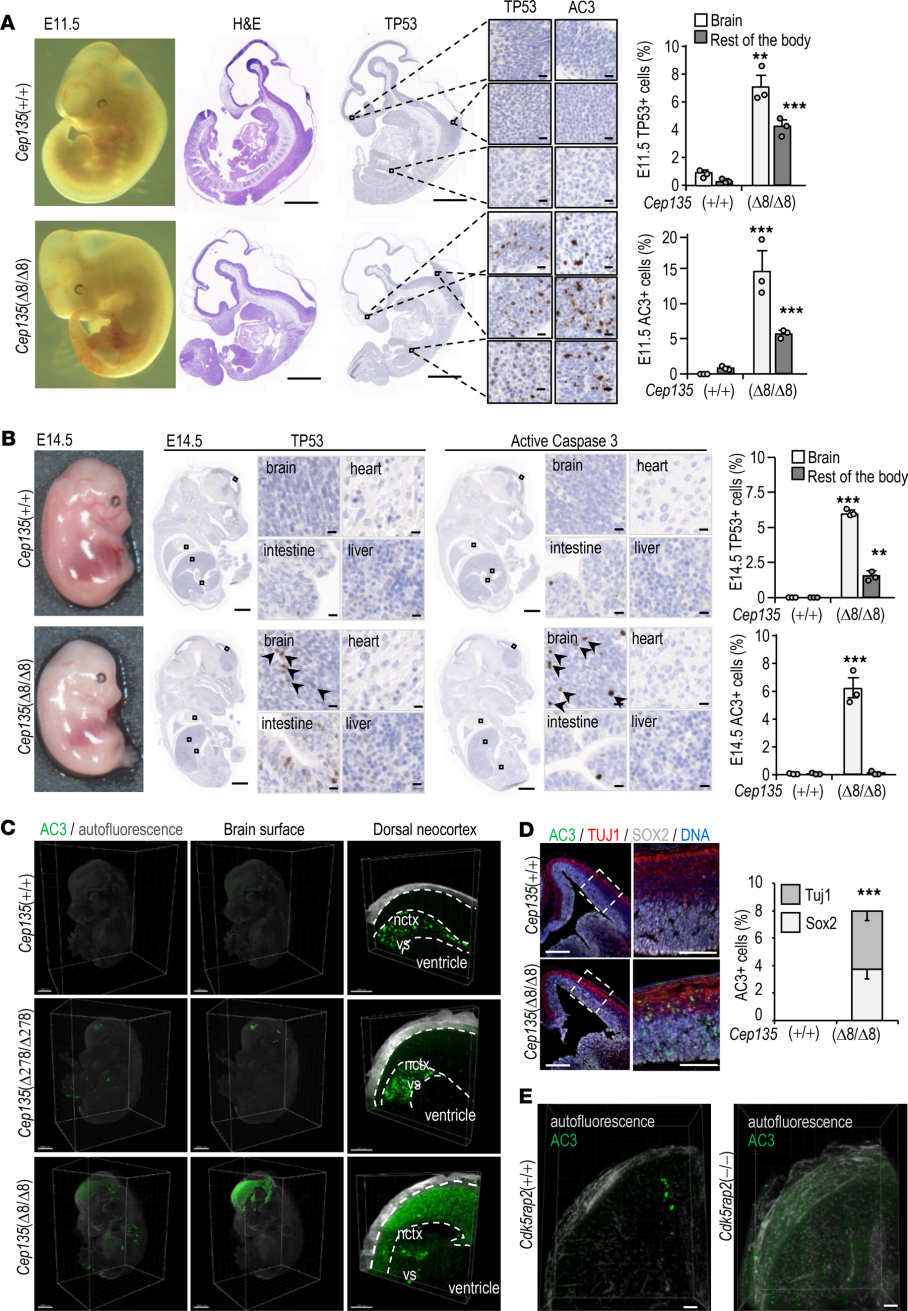
Cep135 deficiency induces TP53-dependent apoptosis in neural progenitors. (**A**) Bright-field macroscopic (left) and H&E staining (middle) images of E11.5 *Cep135*(+/+) and *Cep135*(Δ8/Δ8) mouse embryos. IHC staining for TP53 and active caspase 3 (AC3) in the same samples including insets at higher magnification (TP53, left; AC3, right) from the specific areas (forebrain [upper], medullary hindbrain [middle], and liver [bottom]). The quantification of TP53^+^ and AC3^+^ cells in brain and rest of the body of *Cep135*-mutant and control embryos is shown in the right histograms. Scale bars: 1 mm (whole embryo sections) and 10 μm (insets). (**B**) Bright-field macroscopic images (left), IHC staining (middle), and quantification (right) of TP53^+^ and AC3^+^ cells in brain and rest of the body of E14.5 *Cep135*-mutant and control embryos. Scale bars: 1 mm (whole embryo sections), 100 μm (lower microscopic insets). Representative positive cells are indicated by arrows. (**C**) Whole-mount 3D immunofluorescence of E14.5 *Cep135*(+/+), *Cep135*(Δ278/Δ278), and *Cep135*(Δ8/Δ8) mouse embryos stained for AC3 (green). Gray color depicts tissue autofluorescence in the 488 channel. Scale bars: 1 mm (whole embryos) and 200 μm (lower microscopic insets). Nctx, neocortex; vs, ventricular surface. Note that green, unspecific positive signal in the vs of *Cep135*(+/+) samples corresponds to secondary antibody aggregates. (**D**) Immunostaining for AC3, SOX2, and TUJ1 in 14.5 neocortex from *Cep135*-null and control mice. Scale bars: 250 μm (left) and 100 μm (right). The bottom histogram shows the quantification of AC3^+^ cells in these samples. (**E**) Whole-mount 3D immunofluorescence of E14.5 *Cdk5rap2*(–/–) mouse embryos stained for AC3 (green). Scale bars: 200 μm. In **A**,** B**, and **D**, data are mean SEM from 3 different embryos; **P < 0.01; ***P < 0.001 by Student’s *t* test.

TP53 was also significantly induced in several tissues in E11.5 *Cep135*(Δ8/Δ8) embryos (Figure 5A). This response was accompanied by a significant number of apoptotic cells (as detected by active caspase 3) in the developing brain and the rest of the body, especially dorsal telencephalon cortical cells and hematopoietic progenitors in the embryonic liver. At E14.5, apoptotic cells were observed in the developing neocortex but were almost absent in the rest of the embryonic tissues (Figure 5B). Three-dimensional immunostaining for cleaved caspase 3 in whole embryos suggested that apoptosis was especially evident in the developing neocortex at this stage (Figure 5C). Additional immunofluorescence studies for SOX2 and TUJ1 to detect APs and neuroblasts, respectively, showed apoptotic cell death in both progenitor and neuroblast lineages in the E14.5 developing neocortex (Figure 5D). E11.5 and E14.5 *Cep135*(Δ8/Δ8) embryos also displayed a reduced number of proliferating (as defined by PH3^+^ mitoses) cells in several tissues (Supplemental Figure 6, A and B), suggesting that both early apoptosis and defective proliferation may contribute to reduced body size in these mutant Cep135-deficient embryos. No evidence of apoptotic cells was observed in *Cep135*-mutant embryos at E17.5 (Supplemental Figure 6C), suggesting clearance of apoptotic cells and adaptation to the lack of CEP135 during the later stages of development. Both TP53 and active caspase 3 were also present in *Cdk5rap2*-deficient embryos, whereas these signals were only eventually seen in *Aspm*-deficient embryos (Figure 5E and Supplemental Figure 6, D and E).

### TP53 protects Cep135-mutant brains from structural aberrations during development.

**Figure 6:**
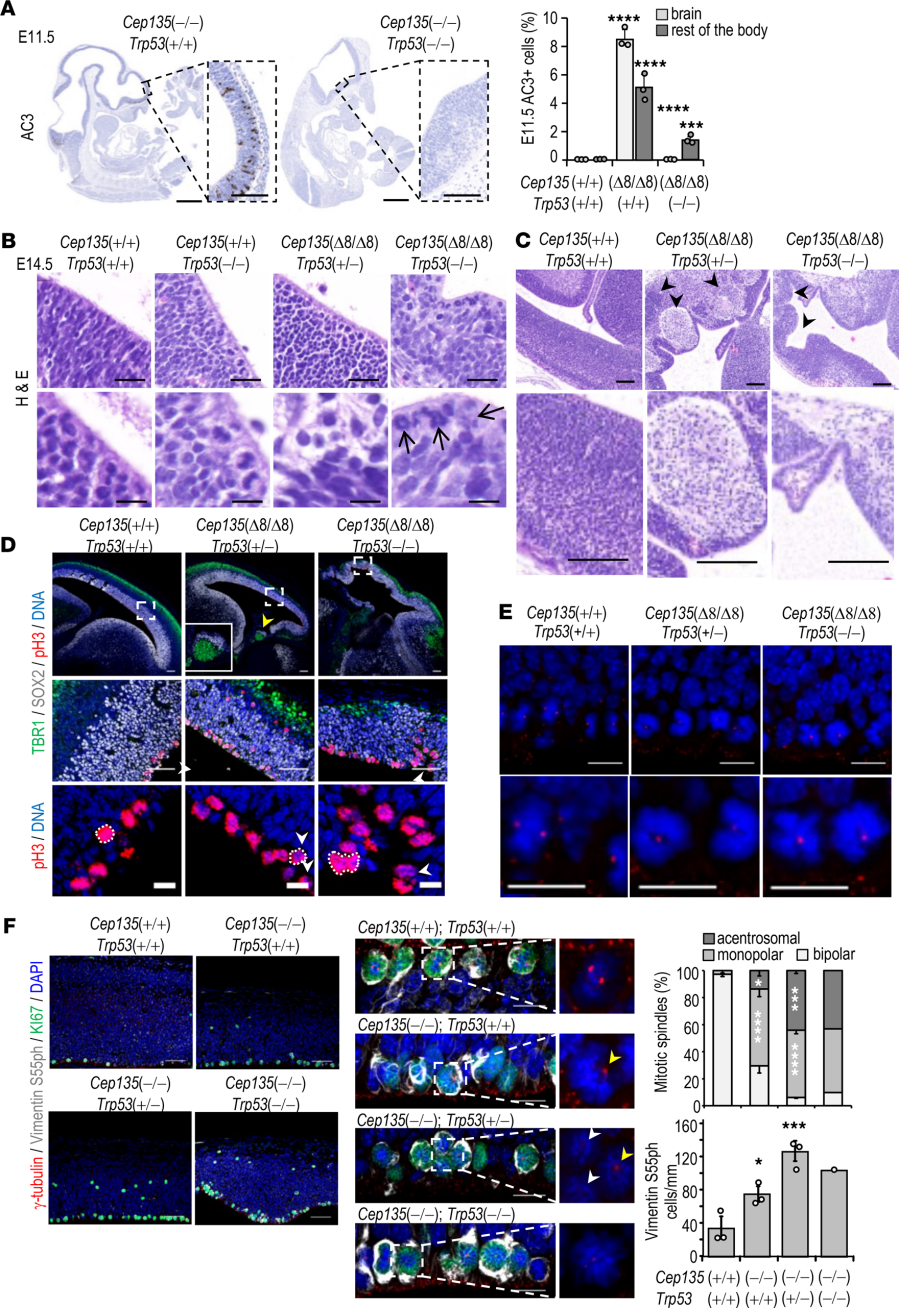
Genetic ablation of Trp53 in Cep135-KO embryos. (**A**) IHC staining for active caspase 3 (AC3; brown) in the indicated models. Scale bars: 1 mm (whole embryos) and 100 μm (insets). In the quantification, data are mean SEM (3 embryos). **P < 0.01; ***P < 0.001 by Student’s *t* test. (**B**) H&E staining of the medial aspect of the neocortex of E14.5 embryos with the indicated genotypes. Scale bars: 20 μm (upper left), 10 μm (lower), and 100 μm (right). Arrows indicate aberrant mitotic figures of apical progenitors. Scale: 10 μm. (**C**) Histological sections of E14.5 neocortices of the indicated genotypes showing cortical heterotopias and malformations (arrowheads). Scale bars: 100 μm (upper) and 50 μm (insets). (**D**) Confocal imaging of E14.5 neocortices stained with the indicated antibodies (upper), and higher magnification confocal images showing the neocortical area delimited by slashed boxes (middle). Yellow arrowhead indicates a subcortical heterotopia. Higher-magnification inset in the middle panel shows CTIP2^+^ heterotopias. Bottom panels show APs with aberrant mitotic figures (arrowheads). Scale bars: 100 μm (upper), 50 μm (middle), and 10 μm (lower). (**E**) Confocal images of APs in the neocortices shown in **D** stained with the indicated antibodies. Scale bars: 10 μm. (**F**) Confocal micrographs of E14.5 neocortices stained with the indicated antibodies. The middle panels show higher-magnification images of the innermost APs with representative mitoses (insets) and monopolar spindles (yellow arrowheads), and acentromal cells (white arrowheads). Scale bars: 50 μm (left), 10 μm (middle), 10 μm (right). Asterisks indicate comparison with *Cep135*(+/+); *Trp53*(+/+) controls. Data are mean SEM from 3 embryos (1 for *Cep135; Trp53* homozygous mutants). *P < 0.01; ***P < 0.001; ****P < 0.0001; 1-way ANOVA with Tukey’s multiple-comparison test.

We next tested the functional relevance of TP53 in the phenotype of *Cep135*-deficient embryos by interbreeding *Cep135*(+/Δ8) with TP53-deficient mice. Lack of TP53 (Supplemental Figure 7A) prevented apoptosis in E11.5 and E14.5 *Cep135*(Δ8/Δ8); *Trp53*(–/–) double mutant embryos (Figure 6A and Supplemental Figure 7A). Intriguingly, TP53 loss did not rescue the reduced thickness of the neocortex in *Cep135*-null embryos (Supplemental Figure 7A). In fact, whereas we did not observe increased lethality of E11.5 *Cep135*(Δ8/Δ8); *Trp53*(–/–) double mutant embryos, we only recovered 1 double mutant embryo alive out of 39 E14.5 embryos derived from 4 matings between *Cep135*(+/Δ8); *Trp53*(–/–) males and *Cep135*(+/Δ8); *Trp53*(+/–) females. Two additional double mutant dead embryos were reabsorbed (Supplemental Figure 7B), suggesting premature embryonic death and embryonic reabsorption at midgestation. Ablation of *Trp53* similarly prevented embryonic development of *Cdk5rap2*-deficient embryos, leading to earlier lethality (~E11.5; Supplemental Figure 7C).

The only E14.5 *Cep135*(Δ8/Δ8); *Trp53*(–/–) double mutant embryo presented profuse MCPH with severe cortical malformations, including abundant aberrant mitotic figures (Figure 6B), as well as perturbed laminar layering of NPs and abnormal neocortical architecture organization typical of subcortical heterotopias (Figure 6C). These abnormalities were accompanied by aberrant mitotic figures in SOX2^+^ progenitors (Figure 6D), as well as monopolar mitosis typical of centrosomal separation defects (Figure 6E). Finally, immunostaining of *Cep135*-mutant neocortices with the mitotic marker phospho–vimentin S55 and the proliferation marker Ki67 showed an increased number of mitotic figures in the ventricular zone (VZ) of *Cep135*(Δ8/Δ8); *Trp53*(+/+) and *Cep135*(Δ8/Δ8); *Trp53*(+/–) double mutant embryos, suggesting that centrosomal defects may be causative of impaired NPC division dynamics during neurodevelopment in these mutant brains (Figure 6F). Additionally, careful examination of the mitotic figures enriched in the *Cep135*(Δ8/Δ8); *Trp53*(+/–) brains revealed an increased frequency of acentrosomal mitoses compared with *Cep135*(Δ8/Δ8); *Trp53*(+/+) brains (Figure 6F), suggesting a role for TP53 in the clearance of these aberrant cells induced by *Cep135* loss during brain development midgestation.

**Figure 7:**
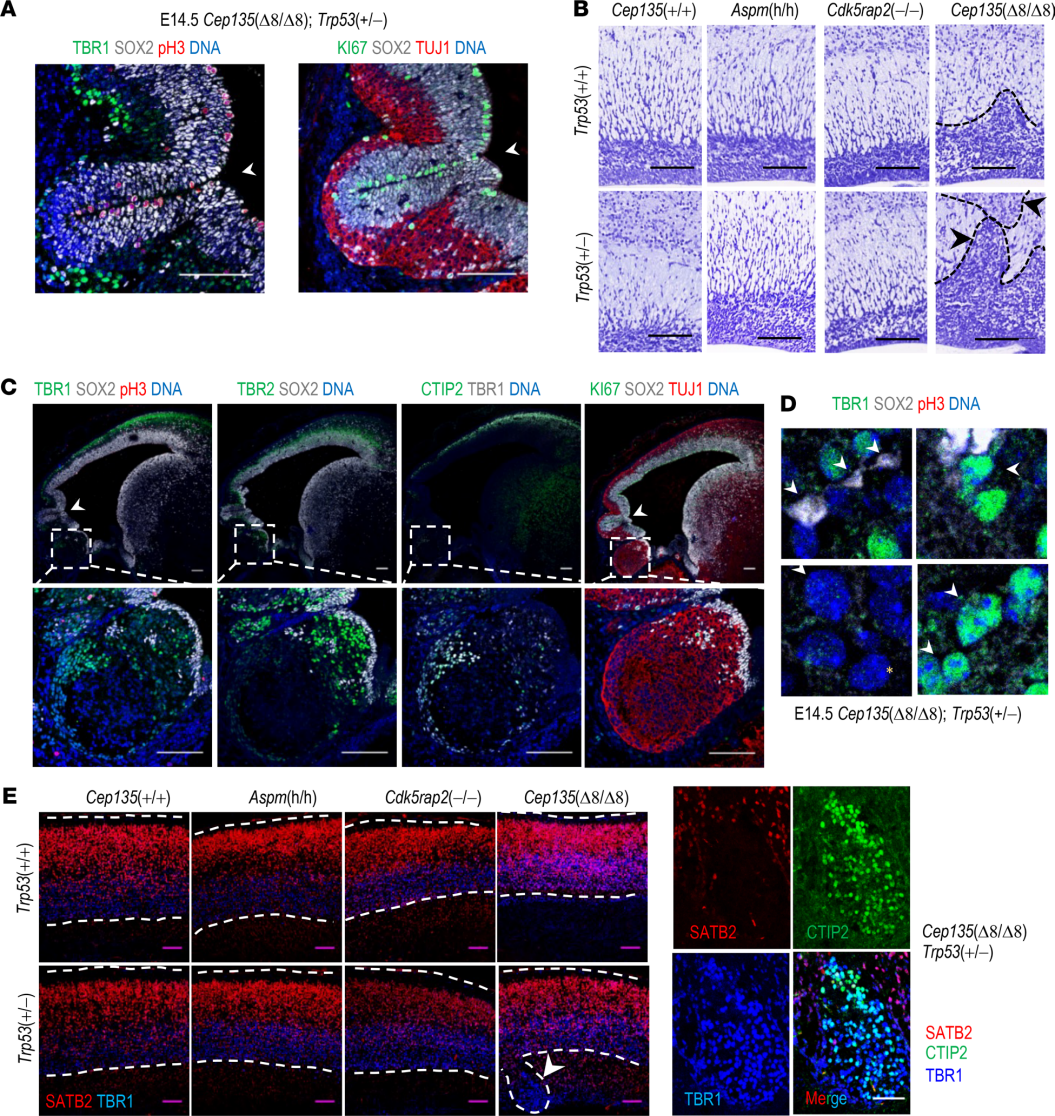
Partial depletion of Trp53 exacerbates the presence of cortical heterotopias in Cep135-mutant newborns but not in other MCPH models. (**A**) Confocal imaging of a E14.5 *Cep135*(Δ8/Δ8); *Tp53*(+/–) neocortex stained with the indicated antibodies. Arrowheads indicate regions of cortical malformations with aberrant folding. Scale bar: 100 μm. (**B**) Histological Nissl staining of P0 cortices from newborns of the indicated genotypes, showing heterotopias in *Cep135*(Δ8/Δ8); *Trp53*(+/–) double mutant newborns (arrowheads). Scale bar: 100 μm. (**C**) Immunostaining of E14.5 embryonic brain in seriated sagittal sections with the indicated antibodies. Arrowheads in upper confocal images depict regions of cortical malformations (also showed in **A**). Squared regions depict a cortical heterotopia, showed in higher magnification in lower panels. Scale bars: 100 μm. (**D**) Higher-magnification confocal images of specific cells from the previous panels showing aberrant and polylobulated nuclei (top arrowheads) and abnormally large nuclei (bottom arrowheads) cells in double mutant heterotopias. A normal nucleus (asterisk) is shown for comparison. Scale bar: 10 μm. (**E**) Immunostaining of P0 neocortices of the indicated genotypes against SATB2 (layers II–V), CTIP2 (layer V), and TBR1 (layer VI). Note the presence of CTIP2^+^, TBR1^+^, SATB2^–^ subcortical heterotopias. Scale bar: 100 μm (left panels) and 50 μm (higher-magnification panels to the right).

Due to the lethality of mutant mice with homozygous mutations in *Trp53* and *MCPH* genes, we also tested the effect of partial ablation of *Trp53* in a MCPH background. Elimination of 1 copy of *Trp53* did not change the ratio of apoptotic cells (data not shown), nor the overall size of the brain in *Aspm*-, *Cdk5rap2*-, or *Cep135-*null newborns (Supplemental Figure 7D). However, *Cep135*(Δ8/Δ8); *Trp53*(+/–) mutant embryos presented cortical malformations (Figure 7A) together with severe cortical dysplasia and subcortical heterotopias within the neocortex (Figure 7B), suggesting that reduced levels of TP53 established a permissive threshold for *Cep135*-deficient cells to form these abnormal clumps of cells. Heterotopias were characterized by the presence of TBR1 and CTIP2 (postmitotic neuroblasts) and TBR2 (IP) positive cells (Figure 7C), a phenotype typically attributed to defects in radial migration of cortical projection neurons in models of autism or related heterotopias (24), as well as the presence of cells with abnormal nuclear morphologies, multilobulated nuclei, or abnormally large nuclei, suggestive of previous aberrations during chromosome segregation (Figure 7D). Interestingly, these heterotopias were characteristic of *Cep135*(Δ8/Δ8); *Trp53*(+/–) mutant embryos and were not observed in double mutants in *Trp53* and other MCPH genes (Figure 7E). Together, these data suggest that CEP135 loss promotes defective centrosome duplication in NPs, leading to TP53-dependent cell death and MCPH, as well as the accumulation of abnormal heterotopias in the presence of reduced TP53 levels.

## Discussion

Despite the identification of more than 20 *MCPH* genes causative of primary MCPH, the cellular basis underlying the neurodevelopmental abnormalities present in patients with most of these specific mutations remains elusive. Interestingly, many *MCPH* gene products are known to be located at the centrosome or spindle poles, pointing to centrosome dysfunction as one of the main causes of MCPH (4, 25). The altered mitotic behavior commonly observed in MCPH usually comprises excessive asymmetric cell division of NPs during brain development, impaired neural differentiation, or cell death of progenitors, which accounts for a reduced pool of progenitors during development, reduced neuronal output, and as a result, MCPH (25, 26).

Biallelic single-bp deletions of the human *CEP135* gene have been reported in MCPH patients with significant MCPH concomitant with severe intellectual disability and communication deficits (17, 27). Previous reports included CEP135 in a group of centriolar proteins, including Sas4 and Sas6, thought to be essential for centriole assembly (8, 9, 25). Several studies have delineated a WDR2/ASPM/SAS4/SAS6 pathway in which these MCPH-associated proteins recruit each other sequentially to the centrosome, thereby enabling centriole duplication to occur (25, 28, 29). CEP135 interacts with SAS6, as well as tubulin, participating in the interaction between the cartwheel and microtubule triplets (30).

Germline ablation of *Sas4* in the mouse results in severe defects in centriole biogenesis and duplication, leading to embryonic death as soon as E8.5 (31). Conditional genetic depletion of these core structural proteins in later stages of embryonic development alter brain neurodevelopment by promoting AP detachment from the ventricular surface, which results in spindle orientation randomization, ectopic proliferation of APs, and progressive loss of centrioles concomitant with TP53 activation and apoptotic death (32). Sas4-null cells lack centrosomes and primary cilia; however, acentriolar spindle poles are assembled, and these mutant cells do not display obvious defects in chromosome segregation (apart from slightly prolonged mitosis) or cell cycle profiles (31). Similarly, genetic ablation of *SAS6* in human cells results in lack of centrioles (33). Whether similar requirements apply to mammalian CEP135, however, is less clear. Pioneering in vitro studies of *Cep135* deficiency using homologous recombination in vertebrate cell lines reported no significant alterations of centriole structure, dynamics, or cell division, although a small decrease in centriole numbers and increase in the frequency of monopolar spindles was observed (15). In contrast to these reports, we observed that complete genetic depletion of *Cep135* in vivo induces significant defects in centriole duplication, leading to frequent apoptotic cell death in several tissues by midgestation. These defects are more pronounced and lasted longer in NPs at later embryonic stages, ultimately leading to MCPH. As opposed to *Sas4*-KO mice, which die before midgestation (31, 32), *Cep135*-deficient mice progressed through embryonic development until birth, indicating that — unlike SAS4 — CEP135 is dispensable for early embryonic development. The abnormal cell division of NPs midgestation in the absence of CEP135 was accompanied by a TP53 transcriptional response and TP53-dependent apoptotic cell death. Ablation of the *Trp53* gene prevented apoptotic cell death but did not rescue the developmental defects caused by *Cep135* ablation. This is in contrast to previous MCPH models in which the developmental delay and MCPH caused by mutations in *Sas4* (31, 32), *Kif20* (34), or *Cep63* (35) were rescued after *Trp53* ablation. The reasons for these differences are unclear but suggest that *Cep135* ablation may alter additional TP53-independent pathways in the developing cortex.

Taken together, these data indicate that CEP135 depletion promotes centrosome duplication defects and mitotic aberrations, accompanied by TP53 activation and defective production of neurons during brain development. The resulting MCPH (25% decrease of cortical area in *Cep135*-mutant newborns) can be considered as severe, taking into consideration the ratio between human and murine encephalization, similar to other genetic mouse models based on *MCPH* gene mutations. The mechanism underlying *Cep135*-null phenotypes — i.e., defective centriole duplication — may, however, be relatively specific. Previous studies have shown that depletion of *Aspm*, which accounts for approximately 60% of human cases of MCPH, in mice promotes mild MCPH and germline defects and affects spindle orientation and astral microtubule dynamics (19, 36, 37). *Cdk5rap2* loss also generates severe MCPH and dwarfism by affecting cell division, causing cell death of NPs during development (21).

Despite the frequency of centrosomal defects characteristic of the perturbed neural development observed in MCPH patients, it remains to be resolved why brain size, in particular, is so vulnerable to these defects (4, 25). Our observations in midgestation embryos suggest that the abnormal divisions and TP53 response to *Cep135* loss are present in additional tissues, such as the embryonic liver. However, TP53 induction and apoptosis is only present in the developing cortex at later developmental stages. It, thus, appears that — compared with other organs — adaptation to centrosome defects is less efficient in the developing neural system, perhaps at least partially explaining the tight correlation between mutation in genes encoding centrosomal proteins and primary MCPH.

Although MCPH does not generally present with gross morphological defects in the neocortex, some cases with structural changes have been reported, including cortical malformations and subcortical heterotopia in patients with mutations in *WDR62* and *CENPJ* (MCPH6) (38, 39). Interestingly, subcortical heterotopia has been recently reported in a *CEP135* patient presenting with epilepsy (18). All these 3 proteins work together in centrosomal microtubule assembly. Interestingly, similar subcortical heterotopias group 1a have been associated with mutations in the genes encoding the katanin subunit KATNB1, also involved in microtubule severing at the centrosome, and the microtubule subunit tubulin β (TUBB), suggesting a link between microtubule assembly and these brain malformations (39).

*Cep135*-deficient mutant embryos do not display heterotopias in the presence of a normal TP53 pathway. As these mutant mice die perinatally, we could not compare this phenotype with the heterotopias observed in the 18-year-old MCPH8 patient (18). However, heterotopias were observed in *Cep135*-mutant embryos with a defective TP53 pathway. Although the functional status of TP53 has not been analyzed in the MCPH8 patient, the *CEP135* mutation was accompanied by other mutations in the same patient, including *FAT4*, a gene previously related to heterotopias. Thus, the causal role of *CEP135* could not be completely established. Multifocal neocortical dysplasias are also common features of autism and underlying causes of epilepsy, which shows strong comorbidity with autism (40). Our data suggest that subcortical heterotopia may arise from centriolar duplication defects in *Cep135*-deficient cells, especially when TP53 is not efficient enough to eliminate aberrant cells during brain development, resulting in a combined phenotype of MCPH and heterotopia. It remains to be tested whether subcortical heterotopia in patients with *CEP135* mutations may arise as a consequence of defective gatekeepers such as TP53 or cooperating mutations (e.g., *FAT4*; ref. 18) that may contribute to the development of this pathology.

## Methods

### Generation of mutant mice.

Two different CRISPR sgRNAs were designed for generating null alleles as follows: *Cep135*_sg#1: 5′-AGTATATTAACATTCGGAAG-3′; *Cep135*_sg#2: 5′-CGGAAGAGGTTAGACCAGCT-3′; *Aspm*_sg#1: 5′-TGGCGACAAAACGGGATTGA-3′; *Aspm*_sg#2: 5′-GGACACGTAGGTCAGCAAAC-3′; *Cdk5rap2*_sg#1 5′-GTCCTTCATGTTCCGGGCTC-3′; *Cdk5rap2*_sg#2: 5′-TTCATGTTCCGGGCTCTGGT-3′. Ribonucleoprotein complexes were assembled by incubating Cas9 protein (LabOmics) (100 ng/mL) and 50 ng/mL of each sgRNA in microinjection buffer (10 mM Tris pH 7.4, 0.1 mM EDTA) for 15 minutes at room temperature (RT). The ribonucleoprotein complexes were injected in the cytoplasm of zygotes obtained from matings of B6.CBA male and female mice at 0.5 days of gestation, using protocols previously described (41). Forty-five zygotes were injected for each model, from which 9, 21, and 18 pups were born for *Cep135*, *Cdk5rap2*, and *Aspm* models, respectively. Genotyping was performed by PCR and sanger sequencing. The genotyping oligonucleotides were as follows: *Aspm* (Fw: 5′-AGTCCCCACCCCATCCATTAG-3′; Rv: 5′-GTTGGCGTATACCCCCGAGT-3′), *Cdk5rap2* (Fw: 5′-ACCACCAAGCAGGAAGTTGC-3′; Rv: 5′-AGTCATGTCAGAGTCCGGGG-3′), *Cep135* (Fw: 5′-TTACTGTCACTAAGGGGTGCC-3′; Rv: 5′-CGTTACTCCCACGCAACTTC-3′), and PCR conditions for the 3 genes indicated were 95°C 5 minutes; (95°C, 30 seconds; 60°C, 30 s; 72°C, 60 seconds) for 40 cycles; and 72°C for 10 minutes. *Trp53*-KO mice were obtained from the Jackson Laboratory (B6.129S2-Trp53tm1Tyj/J; stock no. 002101). Mice were maintained on a C57BL/6J genetic background. For histological examination, samples were fixed in a solution of 10% of buffered formalin (MilliporeSigma), embedded in paraffin, and cut into 2.5 μm sections. The sections were then stained with H&E or Cresyl violet (for Nissl staining).

### Molecular imaging.

Computerized axial tomography scanning (CT scan) was performed on anesthetized P30 mice [3% of Isoflurane (Isoba Vet)] using a PET-CT scan (GE Healthcare) with the following parameters: 200mA, 35 kV, 160 m, 16 shots and 360 projections. MMWKS (GE Healthcare) and MicroView software were used to analyze the resulting images.

### IHC.

Tissues were fixed in 10% buffered formalin (MilliporeSigma) and embedded in paraffin for routine histological analysis. IHC was performed on 2 μm paraffin sections by using an automated protocol developed for the DISCOVERYXT-automated slide-staining system (Ventana Medical Systems Inc.). All steps were performed in this staining platform by using validated reagents, including deparaffinization, acidic antigen retrieval, and antibody incubation and detection. Primary antibodies used for this purpose are listed in Supplemental Table 10. Appropriate biotinylated secondary antibodies were used to detect the primary antibodies, followed by incubation with streptavidin–horseradish peroxidase and diaminobenzidine system. Full slides were digitalized with a Zeiss AxioScan Z1 and analyzed by using the ZEISS Zen 2.3 Imaging Software (Zeiss).

### Immunofluorescence.

Immunohistofluorescence was performed in mouse embryos or newborns tissues. Briefly, mouse embryos or newborns (P0) were decapitated, and brains were dissected out (in the case of P0 newborns). Embryonic heads or P0 brains were fixed in 4% paraformaldehyde (electron microscopy sciences, 50-980-487) for 3 hours at RT and then overnight at 4°C. Next, tissues were rinsed in PBS (MilliporeSigma) 3 times, immersed in 30% sucrose (MilliporeSigma) for 48 hours at 4°C, embedded in Tissue Tek OCT compound (Sakura), and cryosectioned onto SuperFrost Plus slides in a Leica cryostat in cryosections that were 12–14 μm thick. For immunostaining, cryosections were rehydrated and boiled in a microwave oven for epitope retrieval (if indicated) in sodium citrate buffer (10 mM [pH 6]) for 1 minute, maximum. Cryosections were equilibrated in PBS, permeabilized with Triton X-100 0.5% for 5 minutes, washed, and incubated with blocking solution (4% normal goat serum [Vector Laboratories, S-2000] in PBS-T [PBS with Triton X-100 0.1%]) for 1 hour at RT. Primary antibodies listed in Supplemental Table 10 were applied when stated in the indicated concentrations and incubated overnight at 4°C in a humid chamber. Appropriate secondary antibodies (Alexa Fluor dyes from Molecular Probes) were used at a concentration of 1:500 and incubated RT during 3 hours, followed by washing steps, a labeling step with phalloidin–Alexa Fluor 647 (to stain F-actin) or DAPI (to stain nuclei) when indicated. Slides were mounted using Prolong Gold antifade mounting medium or Fluoromount mounting medium, and they were visualized in a Leica TCS SP5 confocal laser microscope using a HCX PLAN APO CS 63×/1.4 oil immersion, HCX PLAN APO CS 40×/1.4 oil immersion, or HCX PLAN APO CS 20×/1.4 objective.

For immunofluorescence of cells in culture, cells were grown onto glass coverslips and fixed in PFA 4% for 15 minutes RT or ice-cold methanol for 5 minutes at –20°C (when indicated); they were then washed in PBS. For neurosphere immunostaining, incipient neurospheres were generated during 2 days in vitro by clonal dilution of primary NPs. Neurospheres were placed onto matrigel-coated glass coverslips (Corning) for 5 minutes and fixed immediately with PFA 4%. Further details about the antibodies used in this study are included in Supplemental Table 10.

### TEM.

Cells were grown on P6MW dishes (Corning) and fixed with glutaraldehyde 4% (MilliporeSigma, G5882). Fixed cells were washed 3× in 0.1M cacodylate buffer (MilliporeSigma); they were then postfixed in 1% osmium tetroxide/1.5% potassium ferrocyanide for 30 minutes. Cells were then washed 3× and incubated in 1% aqueous uranyl acetate (MilliporeSigma) for 30 minutes, washed, and subsequently dehydrated in grades of alcohol (5 minutes each at 50%, 70%, 95%, and 2× 5 minutes at 100%). Cells were embedded in TAAB Epon (Marivac Canada Inc.) and polymerized at 60°C for 72 hours. After polymerization, 1 mm squares of the embedded monolayer were glued onto an empty Epon block for sectioning.

For tissue TEM, E14.5 embryonic brains were dissected and fixed immediately in 2% glutaraldehyde (MilliporeSigma, G5882) 4% paraformaldehyde (Electron Microscopy Sciences, 50-980-487) in 0.4M HEPES buffer for 2 hours at RT. Entire embryonic brains were then postfixed with 1% osmium tetroxide (OsO_4_)/1.5% potassium ferrocyanide (KFeCN_6_) for 1 hour. Then brains were washed in 0.4M HEPES 3× and incubated in 1% aqueous uranyl acetate for 1 hour, followed by 2 washes in 0.4M HEPES and subsequent dehydration in grades of alcohol (10 minutes each at 50%, 70%, 90%, and 2× 10 minutes at 100%). The samples were then infiltrated for 30 minutes in a 1:1 mixture of propylene oxide and TAAB Epon (Marivac Canada Inc.). The samples were embedded in drops of TAAB Epon (Electron Microscopy Sciences) and polymerized in molds at 60°C for at least 72 hours. Ultrathin sections of 40 nm were cut on a Reichert Ultracut-S microtome, placed onto copper TEM grids, stained with uranyl acetate, and examined in a Tecnai G2 spirit transmission electron microscope equipped with a lantane hexaboride (LaB6) filament and a TemCam-F416 (4k × 4k) camera with CMOS technology.

### Cell culture and time-lapse microscopy.

Primary MEFs were isolated from E14.5 mouse embryos, dissociated by trypsinization, and cultured in DMEM with high glucose (MilliporeSigma, D6429) supplemented with 10% FBS (MilliporeSigma), glutamax supplement (Thermo Fisher Scientific, 35050038), and 0.1% gentamycin (Thermo Fisher Scientific). All experiments were performed with primary cultures of passage 0 (at P0).

For time-lapse microscopy, MEFs were seeded in IBIDI 8 well μ-slide chambers (ibidi, 80826) at a medium confluency and nuclei stained for 30 minutes with SiR-DNA kit (Spirochrome) following manufacturer’s instructions. Micrographs in time frames of 10 minutes were acquired during 24 hours in a Deltavision RT imaging system (Olympus IX70/71, Applied Precision) equipped with a Plan apochromatic 20×/1.42 N.A. objective and maintained at 37°C in a humidified CO_2_ chamber. The resulting videos were processed and analyzed with ImageJ software (NIH).

Primary NPs were obtained from E14.5 embryos. Briefly, E14.5 embryonic brains were dissected under aseptic conditions; neocortices from dorsal telencephalon were separated and digested with papain (Worthington, LS00310). Papain was activated in an activation solution consisting of 5.5 mM L-cysteine (MilliporeSigma, C8277) and 1.1 mM EDTA (MilliporeSigma, E6511) in EBSS (Thermo Fisher Scientific, 24010-043) following manufacturer’s instructions, filtered through a 0.22 μM filter and used at a concentration of 12 U/mL (1 mL/2 hemicortices) together with DNAse I (Thermo Fisher Scientific) for 30 minutes at 37°C. NPs obtained were pelleted in DMEM/Hams F-12 (Thermo Fisher Scientific), and activation solution was removed completely. Primary NPs were cultured in suspension in a medium containing DMEM/Hams F-12 (Thermo Fisher Scientific, 11320033), 5 mM HEPES, 1 mM sodium pyruvate, 2 mM L-glutamine, N2 supplement (Thermo Fisher Scientific, 17502048), B27 supplement (Thermo Fisher Scientific, 17504044), 0.7 U/mL heparin sodium salt (MilliporeSigma, H3149), 20 ng/mL mouse EGF (Thermo Fisher Scientific, PMG8044), 20 ng/mL mouse FGF2 (Thermo Fisher Scientific, PMG0034), and penicillin/streptomycin (Thermo Fisher Scientific, 15140148). When needed, neurospheres were dissociated with accutase for 5 minutes at 37°C prior to experiment plating.

### Limiting dilution assays.

Limited dilution assays were performed as previously described (42). Briefly, NP cultures were plated at density of 100 cells/mL and incubated for 48 hours. Neurospheres formed were dissociated and plated in NP medium in 96-well plates at different cellular densities (100, 50, 20, and 10 cells per well). One week later, neurosphere formation was assessed, and wells in which there was at least 1 neurosphere were considered positive. Data in the corresponding representations indicate the fraction of cells with ability to generate cultures with new neurospheres. Graphs were obtained using the ELDA software (42) that processes the data obtained in each experimental condition to the limiting dilution model. In these graphs, the slopes of the depicted solid lines correspond to the fraction of cells with the ability to generate new spheres cultures. A lower slope value indicates a lower fraction of cells with capacity to generate new neurospheres. Dotted lines represent the 95%CI.

### Flow cytometry and metaphase spreads.

Flow cytometry analysis of DNA content was performed by fixing cells with cold 70% ethanol followed by staining with 10 μg/mL propidium iodide (MilliporeSigma). Data acquisition was performed with a Fortessa LSR analyzer (BD Biosciences).

For metaphase spreads, cultured cells were exposed to colcemid (10 μg/mL; Roche, 295892) for 6 hours and pelleted, and individual cell suspension was hypotonically swollen in 50 mL of 75 mM KCl at 37°C for 20–30 minutes. Hypotonic treatment was stopped by gentle addition of 1 mL of Carnoy’s solution (75% pure methanol, 25% glacial acetic acid); cells were then spun down and fixed twice with Carnoy’s solution for 20–30 minutes at RT. After fixation, cells were dropped from a 1 m height onto clean, prewarmed glass slides. Cells were dried overnight at 70°C and stained with Giemsa (MilliporeSigma) afterward, following standard procedures. Images were acquired with a Leica D3000 microscope and a 60× Plan Apo N (numerical aperture, 1.42) objective. Chromosomes from 100 cells per genotype were counted using ImageJ software (NIH).

### Three-dimensional immunofluorescence of E14.5 embryos (FLASH).

Whole E14.5 embryos were processed for FLASH (43) by replacing Borate-SDS with a solution of 3 (dimethyl[tetradecyl]azaniumyl)propane-1-sulfonate detergent (80 g/L), boric acid (200 mM), and urea (250 g/L) (44). Immunofluorescence was performed as stated above using the antibodies indicated in Supplemental Table 10. Images were acquired with a light sheet microscope (LAvision Ultramicroscope II). Selection Plane Illumination Microscopy (SPIM) image 3D reconstruction and surfaces rendering for positive staining was performed with Imaris v9 software (Bitplane).

### RNAseq analysis.

Total RNA derived from E11.5 and E14.5 embryonic neocortices and cultured neurospheres was isolated using mirVana kit (Thermo Fisher Scientific), and sample RNA Integrity was assayed on an Agilent 2100 Bioanalyzer. Sequencing libraries were prepared with the QuantSeq 3′ mRNA-Seq Library Prep Kit (FWD) for Illumina (Lexogen, catalog 015) by following manufacturer instructions. Library generation was initiated by reverse transcription with oligo(dT) priming, and a second strand synthesis was performed from random primers by a DNA polymerase. Primers from both steps contain Illumina-compatible sequences. cDNA libraries were purified, applied to an Illumina flow cell for cluster generation, and sequenced on an Illumina instrument (see below) by following manufacturer’s protocols. Read adapters and polyA tails were removed following the Lexogen recommendations. Processed reads were analyzed with the nextpresso pipeline (45), as follows: sequencing quality was checked with FastQC v0.11.7 (http://www.bioinformatics.babraham.ac.uk/projects/fastqc/). Reads were aligned to the mouse reference genome (GRCm38) with TopHat-2.0.10 using Bowtie 1.0.0 and Samtools 0.1.19 (--library-type fr-secondstrand in TopHat), allowing 3 mismatches and 20 multihits. Read counts were obtained with HTSeq-count v0.6.1 (--stranded=yes), using the mouse gene annotation from GENCODE (gencode.vM20.GRCm38.Ensembl95). Differential expression was performed with DESeq2, using a 0.05 FDR. Enrichr (46) and DAVID (47) were used for gene set enrichment analysis of differentially expressed genes. GSEAPreranked was used to perform gene set enrichment analysis for several gene signatures on a preranked gene list, setting 1000 gene set permutations. Only those gene sets with significant enrichment levels (FDR *q* < 0.25) were considered. Access to RNAseq data are provided from the Gene Expression Omnibus (GEO GSE14749).

### Statistics.

Statistics was performed using Prism software (GraphPad Software) or Microsoft Excel. All statistical tests of comparative data were done using a Mann-Whitney *U* test or an unpaired, 2-tailed Student’s *t* test with Welch’s correction, or 1-way ANOVA with Tukey’s multiple-comparison test when appropriate. Data are expressed as the mean of at least 3 independent experiments ± SEM; a *P* value of less than 0.05 considered statistically significant.

### Study approval.

Mice were housed in a pathogen-free animal facility at the CNIO following the animal care standards of the institution. The animals were observed on a daily basis, and sick mice were humanely euthanized in accordance with the Guidelines for Humane End-points for Animals Used in Biomedical Research (Directive 2010/63/EU of the European Parliament and Council and the Recommendation 2007/526/CE of the European Commission). All animal protocols were approved by the committee for animal care and research of the Instituto de Salud Carlos III/Comunidad de Madrid (Madrid, Spain).

## Author contributions

JGM performed most of the experiments. AWC, DMA, LRLS, JGJ, AM, and AP participated in the analysis of cellular phenotypes and analysis of embryo abnormalities. JA and AB performed the whole-mount immunofluorescence studies. DM, JG, and MPM helped with fluorescent microscopy and JB contributed to TEM studies. OGC helped with the bioinformatics analysis. SO contributed to the injection of mouse embryos. MM designed the project, and JGM and MM wrote the manuscript. All the authors contributed to the analysis of the data.

## Supplementary Material

Supplemental data

Supplemental table 1

Supplemental table 2

Supplemental table 3

Supplemental table 4

Supplemental table 5

Supplemental table 6

Supplemental table 7

Supplemental table 8

Supplemental table 9

## Version 1 07/08/2021

In-Press Preview

## Version 2 08/23/2021

Electronic publication
