# Supplementary material for: Deficient adaptation to centrosome duplication defects in neural progenitors causes microcephaly and subcortical heterotopias
Source: JCI Insight. 2021 Aug 23;6(16):e146364. doi: 10.1172/jci.insight.146364 (PMC8409993; doi:10.1172/jci.insight.146364)
Supplement: Supplemental data [file jciinsight-6-146364-s031.pdf]

Deficient Adaptation to Centrosome Duplication Defects in Neural Progenitors Causes Microcephaly and Subcortical Heterotopias

by José González-Martínez et al.

Inventory of Supplementary Information

Supplementary Figures

Suppl. Figure 1. Generation of *Aspm* and *Cdk5rap2*-mutant mice and analysis of representative pathologies.

Suppl. Figure 2. Generation of *Cep135*-mutant mice and representative pathologies.

Suppl. Figure 3. *Cep135* mutant MEFs present impaired centriole dynamics, increased duration of mitosis, aberrant mitotic spindles and poliploidy.

Suppl. Figure 4. Aberrations in *Cep135*-deficient neural progenitors in the developing neocortex

Suppl. Figure 5. Transcriptomic profiling of *Cep135*-mutant developing brains and cultured neurospheres.

Suppl. Figure 6. *Cep135*-mutant embryos display proliferative defects and TP53-dependent cell death in E11.5 embryos.

Suppl. Figure 7. Loss of TP53 exacerbates the brain defects observed upon *Aspm*, *Cdk5rap2* or *Cep135* depletion.

Supplementary Tables

Suppl. Table 1. Pathways deregulated in E11.5 *Cep135*-deficient cortices.

Suppl. Table 2. Pathways deregulated in E14.5 *Cep135*-deficient cortices.

Suppl. Table 3. Pathways deregulated in E14.5 *Cep135*-deficient cortices cultured for 12 h.

Suppl. Table 4. Pathways deregulated in E14.5 *Cep135*-deficient cortices cultured for 24 h.

Suppl. Table 5. Enrichment in transcription factor binding sites in E11.5 *Cep135*-deficient cortices.

Suppl. Table 6. Enrichment in transcription factor binding sites in E14.5 *Cep135*-deficient cortices.

Suppl. Table 7. Enrichment in transcription factor binding sites in E14.5 *Cep135*-deficient cortices cultured for 12 h.

Suppl. Table 8. Enrichment in transcription factor binding sites in E14.5 *Cep135*-deficient cortices cultured for 24 h.

Suppl. Table 9. Most upregulated genes in *Cep135*-deficient samples.

Suppl. Table 10. Antibodies used in this work.

Supplementary Figures

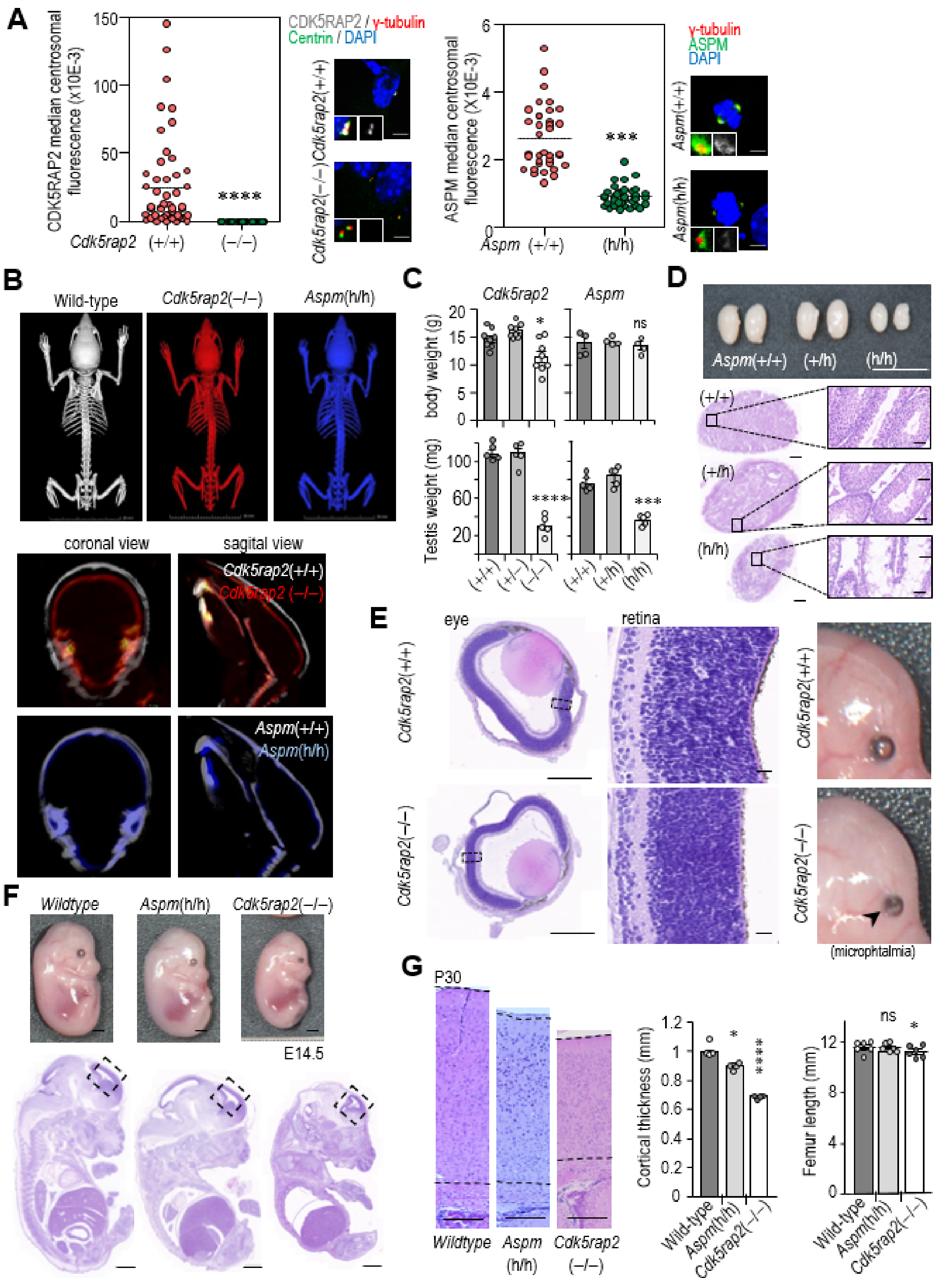

Supplementary Figure 1. Generation of *Aspm*- and *Cdk5rap2*-mutant mice and analysis of representative pathologies (related to Figure 1). A, Confocal imaging of centrosomal loading of CDK5RAP2 (gray, left panels) and ASPM (green, right panels) in MEFs with the corresponding mutant alleles. γ-tubulin is depicted in red, Centrin is in green in the left panels, and DNA (blue) was stained with 4',6-diamidino-2-phenylindole (DAPI). Scale bars: 5 μm. The plots show the

quantification of CDK5RAP2 or ASPM centrosomal protein levels (median fluorescence intensity in arbitrary units) in cells with the indicated genotypes. Horizontal bars depict the mean. B, Micro-CT imaging of wild-type (white), *Cdk5rap2-null* (red) and *Aspm-null* (blue) P30 mice skeletons and skulls in coronal and sagital views. C, quantification of *Cdk5rap2* and *Aspm*-mutant mice body weight (upper histograms) and testis weight (lower histograms). D, Macroscopic image of wild-type and *Aspm*-mutant P30 testis (upper picture) and H&E histological staining of the same testis. Note reduced size and empty seminiferous tubules in *Aspm*(h/h) testis. Micron scale 1cm (upper picture), 100 µm (left panels) and 10 µm (right panels). E, Representative H&E histologies of *Cdk5rap2*-mutant P30 eyes (right panels) with higher magnification insets depicting representative areas of retinal tissue (left panels). Macroscopic images to the left show representative E14.5 *Cdk5rap2*-mutant embryos displaying microphtalmia. Micron scale 1mm (left panels), 50 µm (right panels). F, Macroscopic images (top) and representative H&E histological sections (bottom) of E14.5 *wild-type*, *Aspm* and *Cdk5rap2*-mutant embryos. Slashed boxes delimit the embryonic brain. Scale bar, 1mm. G, Representative H&E sections of coronal sections in the medial aspect of P30 brains of the indicated genotypes. Plots show cortical thickness (as analyzed by histology) and femur length (as analyzed by CT-scan) of P30 mice of the indicated genotypes. Scale bar, 250 µm. In C,G data are mean ± SEM (n=3 embryos per genotype). *, $P<0.05$; ***, $P<0.001$; ****, $P<0.0001$; Student's t-test in A, 1-way ANOVA test with Tukey's multiple comparisons test in C,G.

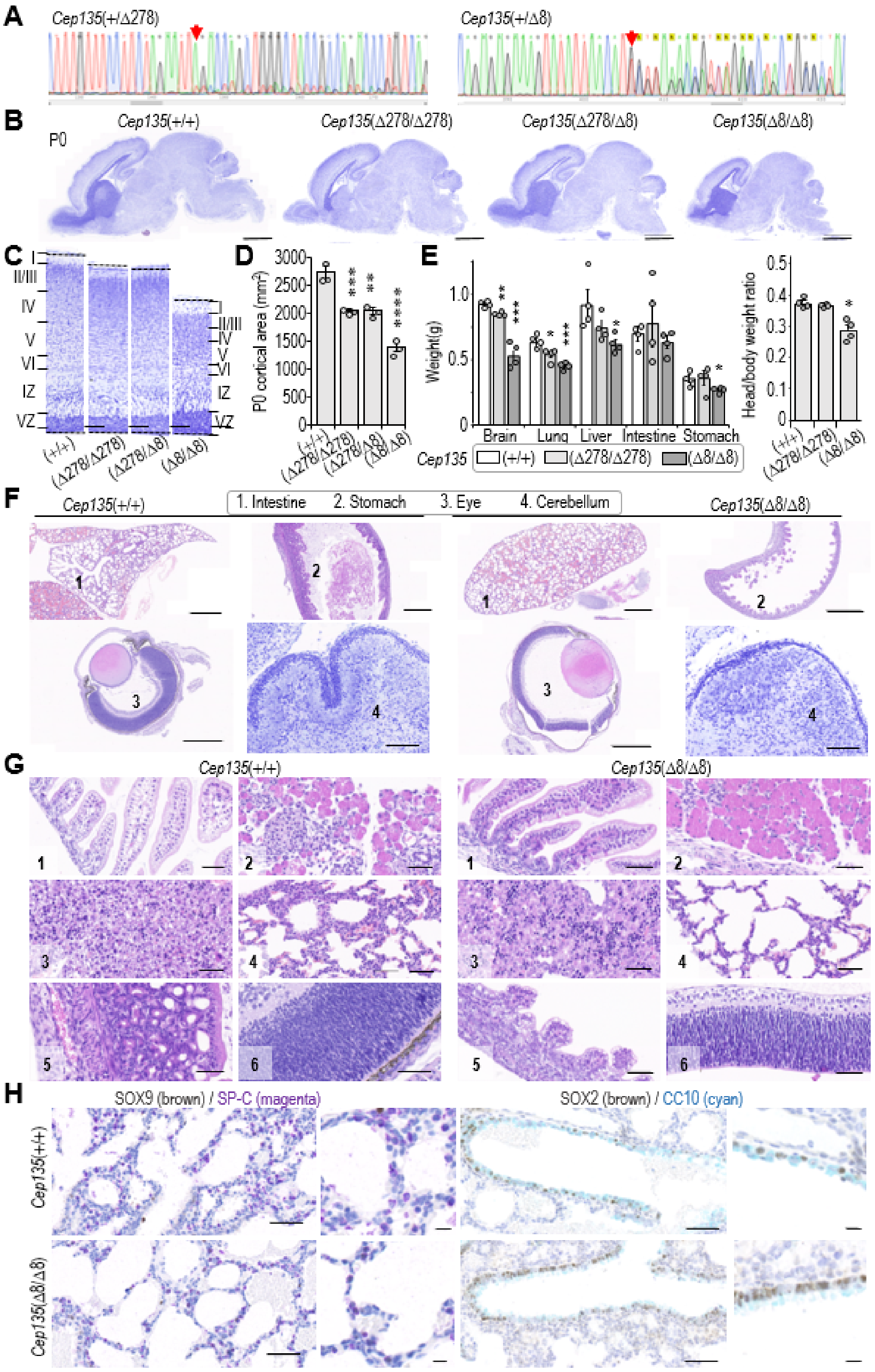

Supplementary Figure 2. Generation of *Cep135*-mutant mice and representative pathologies (related to Figure 1). A, Representative sequences corresponding to *Cep135*(+/Δ278) and

Cep135(+/Δ8) mice. Red arrows indicate indels. B, Representative micrographs of sagital brain sections stained with Nissl at postnatal day 0 (P0) in neonate *Cep135* mutants. Scale bars: 1 mm. C, Histological Nissl staining of P0 cortices from mice with the indicated *Cep135* genotypes. Roman numerals indicate approximate cortical layers in P0 developing brains; IZ: intermediate zone; VZ ventricular zone. Scale bars: 100 µm. D, Quantification of cortical area in brain sections from *Cep135* mutant neonates (P0). E, Quantification of the visceral organs (left) or brain/body (right) weight ratios in P0 pups of the indicated genotypes. F, Histological sections stained with hematoxylin and eosin (H&E, 1-3) or Nissl (4) of the indicated organs of P0 *Cep135*(Δ8/Δ8) and control pups. Scale bars: 1 mm. G, Higher magnification micrographs of *Cep135*(Δ8/Δ8) and control pups viscerae (1, intestine; 2, pancreas; 3, liver; 4, lung; 5, stomach; 6, retina). Scale bars: 100 µm. H, Immunohistochemical staining of *Cep135*(Δ8/Δ8) and control P0 lung sections with the indicated antibodies. Scale bars: 100 µm (micrographs) and 20 µm (higher magnification insets). In D, E, data are mean ± SEM from 4 different mouse pups per genotype; *, $P<0.05$; **, $P<0.01$; ***, $P<0.001$; ****, $P<0.0001$ (1-way ANOVA test with Tukey's multiple comparisons test).

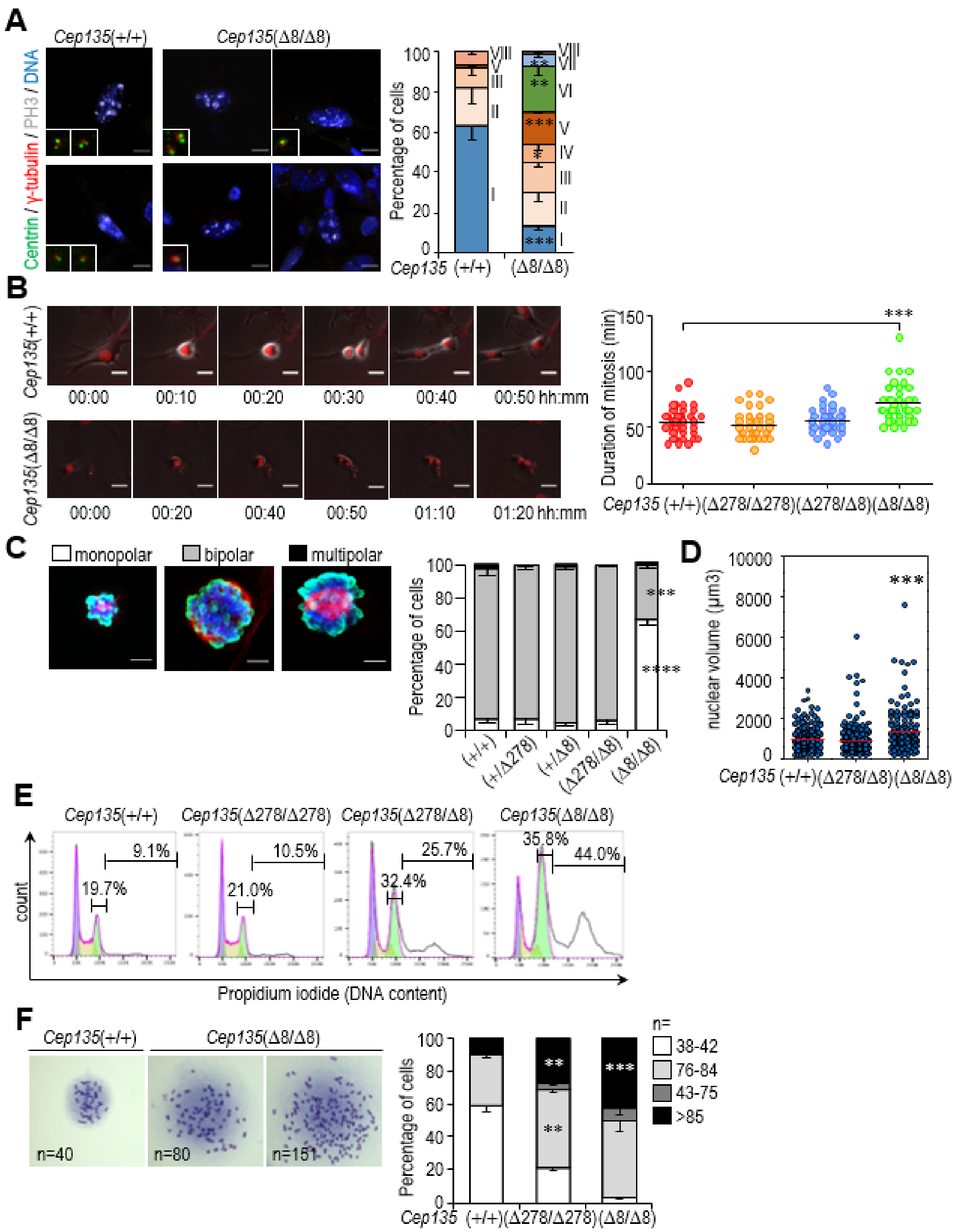

Supplementary Figure 3. *Cep135* mutant MEFs present impaired centriole dynamics, increased duration of mitosis, aberrant mitotic spindles and polyploidy (related to Figure 5). A, Immunostaining for centrin, γ-tubulin and phospho-histone H3 Ser10 (PH3) in E14.5 MEFs of the indicated genotypes. Scale bar: 10 μm. All cells depicted are PH3+. Group I: Two γ-tubulin spots; 2 centrin doublets; II: Two γ-tubulin spots; 2 centrin singlets; III: Two γ-tubulin spots; 1 centrin doublet + 1 centrin singlet; IV: Two γ-tubulin spots; 1 centrin doublet + 1 centrin singlet; V: 1 γ-tubulin spot; 1 centrin doublet; VI: 1 γ-tubulin spot; 1 centrin singlet; VII: acentrosomal; VIII: > 2 γ-tubulin spots. The histogram to the right shows the percentage of cells in each group. B, Time-lapse imaging of E14.5 wild-type and *Cep135*(Δ8/Δ8) dividing MEFs. Representative insets depict

micrographs for different time points detailed below. The histogram to the right depicts the duration of mitosis (DOM) for the indicated genotypes. Scale bar: 10 µm. C, Representative confocal images of monopolar, bipolar and multipolar mitotic spindles in cells stained with the indicated antibodies. The histogram to the bottom depicts the percentage of each mitotic spindle type per genotype. Scale bar: 10 µm. D, Quantification of nuclear volume of E14.5 MEFs from the indicated genotypes. E, Cell cycle profiles showing DNA content in E14.5 MEFs of the indicated genotypes. Horizontal lines delimitate the percentage of each population of cells according to their DNA content. F, Representative pictures of metaphase spreads of E14.5 *Cep135*(∆8/∆8) MEFs and quantification of groups of cells depending on the chromosomic numbers. n indicates number of chromosomes per cell: euploid (2n=40)/tetraploid (4n=80) or near euploid/tetraploid (38-42 and 76-84), or aneuploid (43-75 or >85). In A,C,F data are mean ± SEM; n>150 cells from three independent experiments in a, n>50 cells in B and F, and n>100 cells in C. In B,D horizontal lines represent mean. In A, **, *P*<0.01; ***, *P*<0.001 (Student's t-test). In B,C,D,F, ns, non-significant; *, *P*<0.05; **, *P*<0.01; ***, *P*<0.001; ****, *P*<0.0001; 1-way ANOVA test with Tukey's multiple comparisons test.

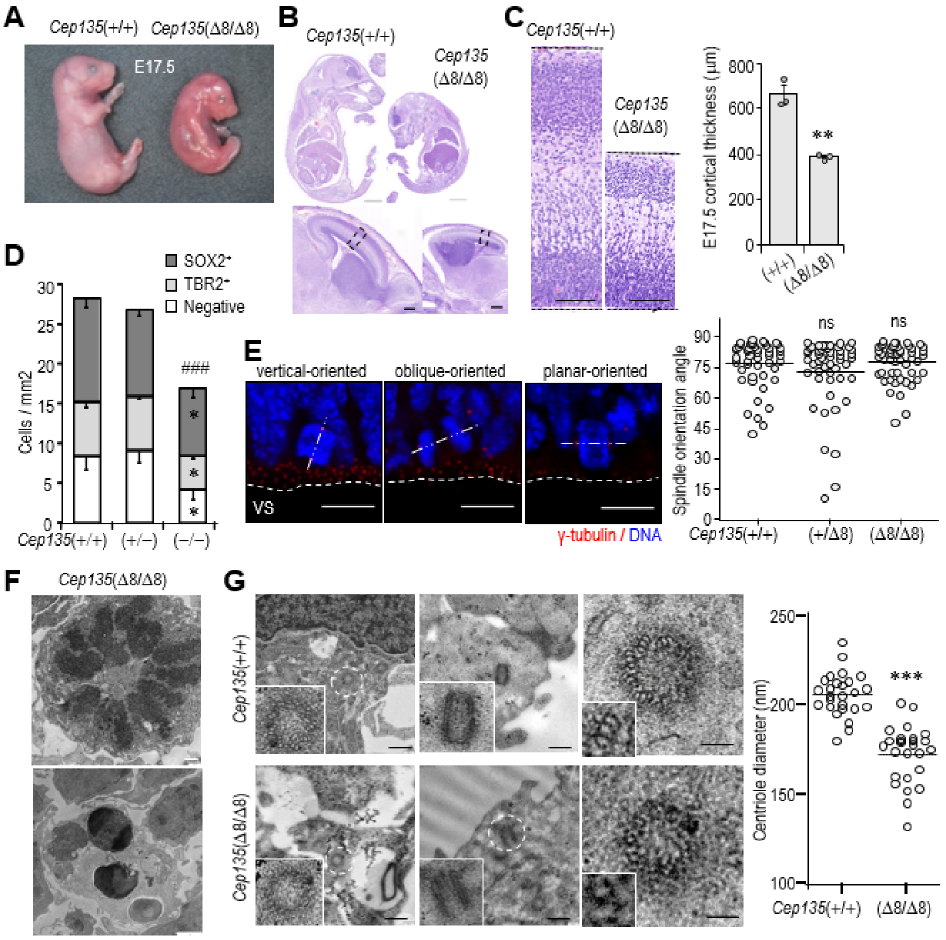

Supplementary Figure 4. Aberrations in *Cep135*-deficient neural progenitors in the developing neocortex (related to Figure 3). A, Representative macroscopic images of *Cep135*(+/+) and *Cep135*(Δ8/Δ8) E17.5 fetuses. B, Haematoxylin and eosin (H&E) staining of histological sagital sections from E17.5 *Cep135*-null and control fetuses. Scale bars: 1 mm (top) and 500 μm (bottom panel showing a higher magnification of the brain area). C, Representative images of H&E-stained sections of cortices from these E17.5 fetuses. Scale bars: 100 μm. The histogram to the right shows the quantification of cortical thickness of the medial aspect in these neocortices. **, *P*<0.01 (Student's t-test). D, Quantification of NPCs positive for the indicated markers per area units in E14.5 embryonic neocortices. Asterisks indicate statistical comparison between each group and *Cep135*(+/+); # indicates comparison between the total number of cells/micrograph area versus *Cep135*(+/+) samples. E, Representative confocal micrographs of dividing neural progenitors in the ventricular surface (VS) of E14.5 embryos stained for γ-tubulin (red) and DNA (blue). Slashed lines decorating the apical side of γ-tubulin dots represent the VS; dashed and dotted straight lines linking the two centrosomes in each picture represent the approximate orientation of the mitotic spindle. Scale bar: 10 μm. The plot to the bottom depicts the mitotic spindle orientation angle respect the VS; horizontal lines represent mean. F, Representative transmission electron microscopy images of monopolar spindles (left) and apoptotic bodies in *Cep135*(Δ8/Δ8) mutant cells in the developing neocortex. Scale bars: 500 nm (left) and 2 μm (right). G, Representative transmission electron microscopy images of centrioles in the ventricular surface tissue of wild-type and *Cep135*(Δ8/Δ8) E14.5 embryos. Scale bars: 10 nm. The quantification of centriole diameter is shown to the right. ***, *P*<0.001 (Student's t-test). In D,E, ns, non-significant; *, *P*<0.05; ***, *P*<0.001; 1-way ANOVA test with Tukey's multiple comparisons test.

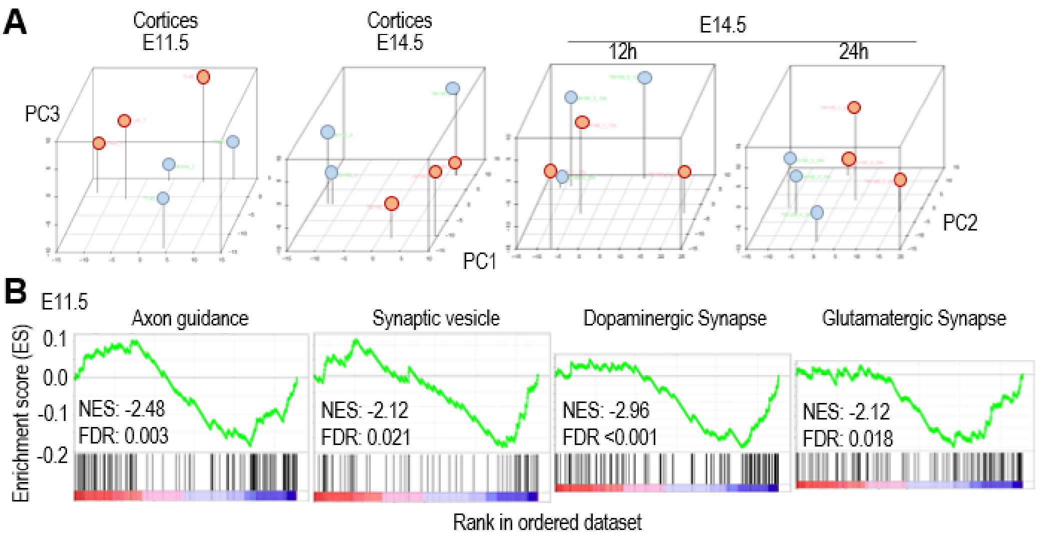

C

E14.5 – 0h

Deregulated pathways		*Enrichment in TF targets*	
Cluster of Pathways	*Enrichment Score*	Enriched term	*p* value
Infection and complement	1.3785	SUZ12 Chea	0.000
Regulation of transcription	0.9462	AR Chea	0.001
Apoptosis	0.8141	Rest Chea	0.003
Protein phosphorylation	0.3810	TP53 Chea	0.004
G-protein coupled receptors and signal transduction	0.3144	TP64 Chea	0.033

E14.5 – 12h

Deregulated pathways		*Enrichment in TF targets*	
Cluster of Pathways	*Enrichment Score*	Enriched term	*p* value
Nervous system differ.	2.1195	SUZ12 Chea	0.000
cAMP signaling & synapsis	1.6498	SMAD4 Chea	0.001
Cell cycle	1.6477	SUZ12 ENCODE	0.009
Ion transport	1.6048	Rest Chea	0.010
Synapses signaling	1.5809	GATA2 Chea	0.017

Supplementary Figure 5. Transcriptomic profiling of *Cep135*-mutant developing brains and cultured neurospheres (related to Figure 4). A, Principal component analysis of the transcriptomic profiles from the indicated *Cep135*(Δ8/Δ8) or *Cep135*(+/+) samples (see Figure 2 for details). B, GSEA analysis of major pathways deregulated in E11.5 *Cep135*(Δ8/Δ8) samples. C, Major pathways deregulated and enrichment in transcription factor (TF) targets in E14.5 samples before (0 h) and after being cultured during 12 h to form neurospheres. See Supplementary Tables 1-9 for additional details.

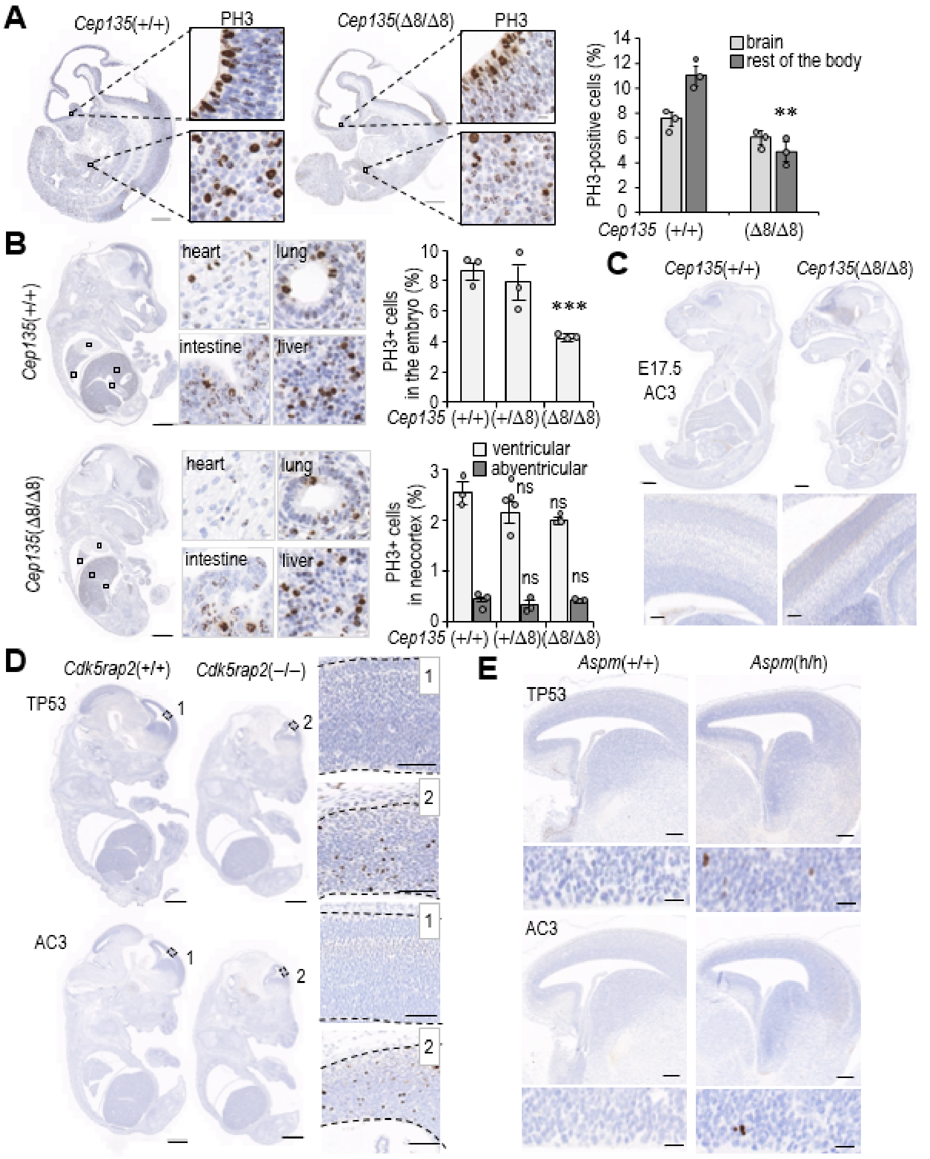

Supplementary Figure 6. *Cep135* mutant embryos display proliferative defects and TP53-dependent cell death in E11.5 embryos (related to Figure 5). A, Immunohistochemical staining of phospho-histone H3 Ser10 (PH3) in wild-type and *Cep135*(Δ8/Δ8) E11.5 mouse embryos. The histogram to the right shows the percentage of mitotic PH3+ (excluding G2 cells) cells in each group. Scale bars: 500 µm (whole embryo sections), 10 µm (insets). B, Immunohistochemical staining of PH3 in E14.5 mouse embryos of the indicated genotypes. Scale bars: 1 mm (whole embryo sections), 10 µm (insets). Percentage of PH3+ cells in the embryonic body (top histogram), and percentage of ventricular and abventricular mitoses in the dorsal neocortex (bottom). C, Immunohistochemical staining of active caspase 3 (AC3) in E17.5 fetuses of the indicated genotypes. Insets depict higher magnification micrographs of the medial aspect of the dorsal cortex. Scale bars: 1 mm (whole embryo sections), 100 µm (insets). D, Immunohistochemical staining of TP53 and AC3 in E11.5 embryos of the indicated genotypes. Scale bars: 1 mm (whole embryo sections), 100 µm (insets). E, Representative bright-field macroscopic images of four E14.5 embryos within two litters resulting from matings between *Cep135*(+/Δ8); *Trp53*(–/–) males and *Cep135*(+/Δ8); *Trp53*(+/–) females. Arrowheads indicate *Cep135*(Δ8/Δ8); *Trp53*(–/–) embryos being reabsorbed. In A,B data are mean SEM from 3 different embryos per genotype group; ns, non-significant; **, *P*<0.01; ***, *P*<0.001; Student's t-test (A) and 1-way ANOVA test with Tukey's multiple comparisons (B).

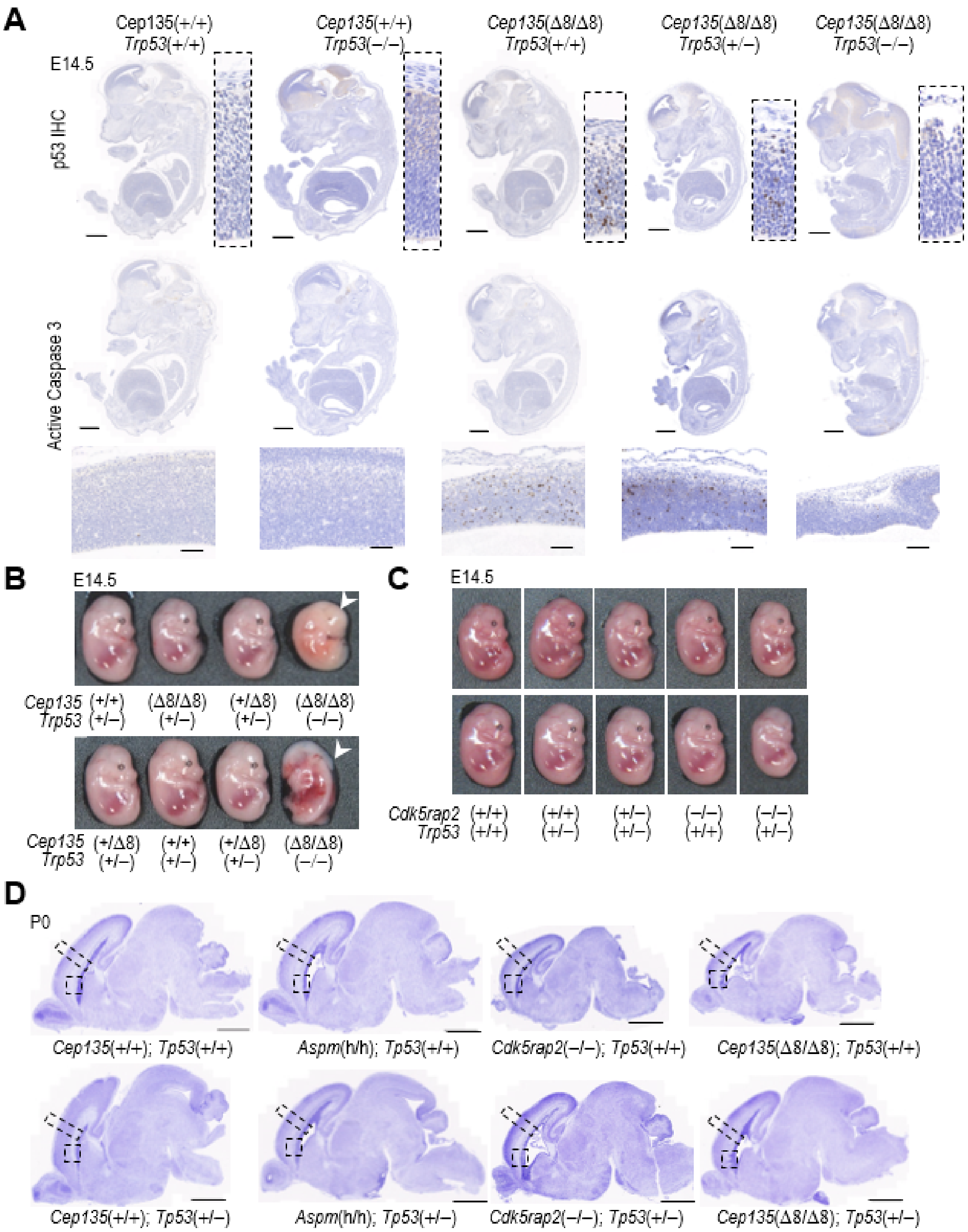

Supplementary Figure 7. Loss of TP53 exacerbates the brain defects observed upon *Aspm*, *Cdk5rap2* or *Cep135* depletion (related to Figure 7). A, Immunohistochemical staining for p53 or active caspase 3 (brown, whole embryos and insets) in *Cep135*(+/+) and *Cep135*(Δ8/Δ8) E14.5 embryos in a *Trp53*(+/+), *Trp53*(+/–) and *Trp53*(–/–) genetic background. Scale bars: 1 mm, 100 μm (insets). B, Representative bright-field macroscopic images of four E14.5 embryos within two litters resulting from matings between *Cep135*(+/Δ8); *Trp53*(–/–) males and *Cep135*(+/Δ8); *Trp53*(+/–) females. Arrowheads indicate *Cep135*(Δ8/Δ8); *Trp53*(–/–) embryos being reabsorbed. C, Bright-field macroscopic images of five E14.5 embryos within two litters resulting from matings between *Cdk5rap2*(+/–); *Trp53*(–/–) males and *Cdk5rap2* (+/–); *Trp53*(+/–) females. D, Representative micrographs of sagital brain sections stained with Nissl at postnatal day 0 (P0) in neonate mutants for the indicated genotypes. Scale bars: 1 mm.

Supplementary Tables

Supplementary Table 1. Pathways deregulated in E11.5 *Cep135*-deficient cortices (see Excel file).

Supplementary Table 2. Pathways deregulated in E14.5 *Cep135*-deficient cortices (see Excel file).

Supplementary Table 3. Pathways deregulated in E14.5 *Cep135*-deficient cortices cultured for 12 h (see Excel file).

Supplementary Table 4. Pathways deregulated in E14.5 *Cep135*-deficient cortices cultured for 24 h (see Excel file).

Supplementary Table 5. Enrichment in transcription factor binding sites in E11.5 *Cep135*-deficient cortices (see Excel file).

Supplementary Table 6. Enrichment in transcription factor binding sites in E14.5 *Cep135*-deficient cortices (see Excel file).

Supplementary Table 7. Enrichment in transcription factor binding sites in E14.5 *Cep135*-deficient cortices cultured for 12 h (see Excel file).

Supplementary Table 8. Enrichment in transcription factor binding sites in E14.5 *Cep135*-deficient cortices cultured for 24 h (see Excel file).

Supplementary Table 9. Most upregulated genes in *Cep135*-deficient samples (see Excel file).

Supplementary Table 10. Antibodies used in this work.

Antigen	Species	Uses and dilution	Origin
α-tubulin	mouse (IgG1)	IF (1:1000)	Sigma (T9026)
ASPM	rabbit	IF (1:500)	W. Huttner, MPI-CBG, Dresden. Immunogen: mAspm exon 3 peptide
CC10	goat	IF (1:200)	Santa-Cruz (sc-9772)
CDK5RAP2	rabbit	IF (1:500-1000)	Millipore (06-1398)
Cenexin/ODF2	rabbit	IF (1:200)	Abcam (ab43840)
Centrin	mouse (IgG2a)	IF (1:500)	Millipore (04-1624)
CEP135	rabbit	IF (1:100)	Abcam (ab75005)
Cleaved-caspase 3	rabbit	IF (1:100), IHC (1:200)	CST (9661S)
Cyclin A	rabbit	IF (1:250)	Santa-Cruz (sc-751)
γ-tubulin	mouse (IgG1)	IF (1:1000)	Sigma (T6557)
Histone H3 (phospho Ser10)	mouse (IgG1)	IF (1:500)	Millipore (05-806)
TP53	mouse (IgG1)	IF (1:200)	CST (2524)
PCNA	mouse (IgG2a)	IF (1:200)	Millipore (NA03)
SAS6	mouse (IgG2b)	IF (1:250)	Santa-Cruz (sc-81431)
SOX2	goat	IF, IHC (1:200)	R&D systems (AF2018)
SPC	rabbit	IHC (1:100)	Millipore (AB3786)
TBR2	rabbit	IF (1:250)	Abcam (ab23345)
TUJ1	Mouse (IgG2a)	IF (1:250)	Covance (MMS-435-P)
Vimentin (phospho Ser55)	mouse (IgG2b)	IF (1:200)	Abcam (ab23345)
CTIP2	rat	IF (1:100)	Abcam (ab18465)
SATB2	mouse (IgG1)	IF (1:500)	Abcam (ab51502)
TBR1	rabbit	IF (1:100)	Abcam (ab31940)
KI67	rabbit	IF (1:500)	Abcam (ab16667)
